# Targeted inhibition of Ninjurin2 promotes chemosensitivity in chemoresistant gastric cancer by suppressing cancer-initiating cells

**DOI:** 10.1186/s40364-025-00792-0

**Published:** 2025-06-15

**Authors:** Hyo Shik Shin, Jae-Il Choi, Hye Won Chung, Hee Jung Park, Hak Park, John Hoon Rim, Jong-Baeck Lim

**Affiliations:** 1 Department of Laboratory Medicine, Yonsei University College of Medicine, Seoul, Republic of Korea; 2 Department of Pathology, Ajou University School of Medicine, Suwon, Republic of Korea; 3 Department of Pathophysiology, College of Basic Medical Science, China Medical University, Shenyang, China

**Keywords:** Ninjurin2, Chemoresistance, Gastric cancer cells, Cancer initiating cells, Organoid

## Abstract

**Background:**

The combination of epirubicin, cisplatin, and 5-fluorouracil (ECF) is widely used for gastric cancer treatment. However, cancer cells can acquire chemoresistance over multiple treatment cycles, leading to recurrence. This study aimed to investigate a novel biomarker for predicting ECF resistance and its biological roles in gastric cancer.

**Methods:**

ECF-resistant (ECF-R) gastric cancer cell lines were established through stepwise ECF treatment. Transcriptome analysis was performed to identify resistance-related genes, which were validated in tumor organoids and in vivo models. Additionally, gastric cancer patient tumor tissues were analyzed for clinical relevance.

**Results:**

Transcriptome analysis revealed that *NINJURIN2* and *CD44* were highly expressed in ECF-R cells but rarely expressed in normal gastric tissues. NINJURIN2 inhibition significantly increased chemosensitivity to ECF in vitro and in vivo. Liquid chromatography–tandem mass spectrometry identified periostin as a binding partner of NINJURIN2, mediating chemoresistance. Furthermore, VAV2 phosphorylation was markedly upregulated in ECF-R cells but was inhibited by NINJURIN2 knockdown. Clinical analysis showed that high NINJURIN2 expression correlated with poor survival outcomes in gastric cancer patients.

**Conclusion:**

Our findings suggest that NINJURIN2 can be used as a novel biomarker for chemoresistant gastric cancer patients and that inhibiting NINJURIN2 along with standard chemotherapy could prevent chemoresistance-associated relapse in gastric cancer.

**Supplementary Information:**

The online version contains supplementary material available at 10.1186/s40364-025-00792-0.

## Background

In 2020, gastric cancer was the fifth most frequently diagnosed cancer and the third leading cause of cancer-related deaths worldwide [1–3]. According to the Lauren classification system, gastric cancer can be classified into two main types: diffuse and intestinal [4]. The relative incidence rates of gastric cancer types are approximately 54%, 32%, and 15% for intestinal, diffuse, and indeterminate, respectively. The intestinal type is primarily associated with environmental factors, such as *Helicobacter pylori* infection, diet, and lifestyle. In contrast, the diffuse type is more commonly associated with genetic abnormalities [5].

The 5-year survival rate for patients with gastric cancer has improved with advances in early diagnosis and treatment. Perioperative chemoradiotherapy has substantially increased survival rates compared with surgery alone [6]. According to the 2019 National Comprehensive Cancer Network (NCCN) guidelines, a primary chemotherapy regimen for metastatic gastric cancer includes a combination treatment of epirubicin, cisplatin, and 5-fluorouracil (ECF), with paclitaxel and irinotecan used as the second-line chemotherapy. The ECF combination treatment was first developed by the Royal Marsden Hospital in 1991 [7]. In the Medical Research Council Adjuvant Gastric Infusional Chemotherapy trial, the perioperative ECF treatment group had a better 5-year survival rate (36%) than the surgical treatment group (23%) [6]. Each drug in this regimen was selected based on its individual efficacy and synergistic effects with the other two agents [7]. However, chemoresistance remains a significant obstacle to effective gastric cancer treatment.

Generally, chemoresistance can be divided into two subtypes: intrinsic resistance, which is caused by pre-existing resistance-mediating factors, and acquired drug resistance, which develops during chemotherapy [8]. Cancer cells develop chemoresistance through mechanisms such as increased drug efflux, mutations in drug targets, DNA damage repair, activation of alternative signaling pathways, and evasion of cell death [8]. Most of the current chemotherapeutic strategies target cancer cell proliferation. In quiescent and slow-cycling cancers, cancer-initiating cells (CICs) can evade standard anticancer therapeutics such as cisplatin, eventually leading to cancer relapse [9]. Previous experimental investigations have led to the development of drugs that target CIC-related pathways in gastric cancer, such as CD44 inhibitors, C-MET (HGFR) inhibitors, and Notch and mTOR inhibitors [10]. However, recipients of these antigen-targeted therapies have reported side effects [10] that occur mainly because the targeting antigens are widely expressed in normal tissues, complicating efforts to selectively target cancer cells.

Nerve injury-induced protein 2 (Ninjurin2, NINJ2), a member of the family of ninjurins, is a surface adhesion molecule that is expressed in mature sensory and enteric neurons. Under pathological conditions, NINJ2 is expressed in Schwann cells of injured nerves [11] and is associated with an increased risk of ischemic stroke [12]. NINJ2 also induces hepatic fibrosis in hepatocytes by activating the IGF1R/EGR1/PDGF-BB signaling pathway [13]. Additionally, the knockdown of NINJ2 in human vascular endothelial cells can decrease the expression of atherosclerosis-related markers and chemokines, especially those related to endothelial cell activation and inflammation [14]. Recent evidence suggests that NINJ2 plays an oncogenic role in tumor progression. NINJ2 expression is significantly upregulated in both glioma and human colorectal cancer, promoting cell growth via activation of the Akt and ERK signaling pathways [15, 16]. Although NINJ2 has been implicated in various human diseases, including cancer, the specific mechanisms underlying its biological roles and potential oncogenic functions remain poorly understood.

The aim of this study was to investigate the mechanisms underlying resistance to ECF chemotherapy in gastric cancer, focusing on the role of the NINJ2 protein. To define the role of NINJ2 in chemoresistance, CIC enrichment, and clinical outcomes, we developed ECF-resistant (ECF-R) gastric cancer cell lines and organoids. Moreover, we explored whether targeting NINJ2 could enhance chemosensitivity and reduce relapse, providing potential for NINJ2 as a biomarker and therapeutic target to improve treatment outcomes for patients with gastric cancer.

## Methods

### Reagents

Epirubicin, cisplatin, and 5-fluorouracil (5-FU) were purchased from Sigma Aldrich (St. Louis, MO, USA).

### Generation of chemo-resistant gastric cancer cell lines

The gastric cancer cell lines were treated with the IC_50_ concentration of ECF, which varied by cell line. After 72 h, the media were changed to drug free media, and we performed sub-culturing twice. Subsequently, each cell line was exposed to the appropriate IC_70_ and IC_80_ concentrations, and then we repeated those procedures. To prevent reversion to the chemo-sensitive equilibrium, we treated the gastric cancer cell lines with the appropriate IC80 concentration of ECF every 2 weeks. All experiments were performed after sub-culturing twice.

### RNA sequencing

For RNA isolation, the total RNA of parental and ECF-R MKN-28/74 gastric cancer cells was isolated using an RNA isolation kit (Qiagen, Hilden, Germany). The mRNA was isolated from the total RNA using a Poly(A) RNA selection kit (LEXOGEN, Austria), and cDNA was synthesized following the manufacturer’s protocol. Libraries were checked using an Agilent 2100 bioanalyzer (DNA high sensitivity kit, Agilent Technologies, Amstelveen, Netherlands) to evaluate the mean fragment size. High-throughput sequencing was performed after quantification using a HiSeq 2500 (Illumina, San Diego, CA, USA) and paired-end 100 sequencing.

To obtain alignment file, the mRNA-Seq reads were mapped using the TopHat software tool [17]. Coverage in Bedtools was used to evaluate differentially expressed genes based on counts from unique and multiple alignments [18]. The read count data were recorded the using the quantile normalization method and EdgeR. The alignment files were also used to assemble transcripts, estimate abundance, and detect differential expression of genes or isoforms through cufflinks. Gene lists associated with a specific functional GO (Gene Ontology) annotation were identified using the gene ontology resource [19].

### Phospho-protein antibody array

To investigate NINJ2 signaling, we examined a phospho-protein antibody array (PEX100, Fullmoon Biosystems, Sunnyvale, CA, USA) containing 1,318 site-specific and phospho-specific antibodies, with 2 replicates per antibody, in NINJ2-overexpressing MKN-74 cells. The phospho-protein antibody array was performed according to the manufacturer’s instructions (Fullmoon Biosystems). Briefly, protein from control cells and NINJ2 iso-1- and iso-3-overexpressing MKN-74 cells were extracted using a protein extraction buffer. Then the protein solution was purified using a gel matrix column. The purified protein was mixed with labeling buffer and treated with a biotin/dimethyl formamide solution. The labeled sample was incubated and blocked on an array slide on a shaker at 60 rpm for 2 h at room temperature. After incubation, the slide was washed 6 times on a shaker at 60 rpm for 5 min each time. For detection, the slide was incubated with Cy3-streptavidin on the shaker at 60 rpm for 20 min. The slide was then washed 6 times with washing solution. For scanning, we used a GenePix 4100 A scanner (Axon Instrument, Sunnyvale, CA, USA). Scan images were quantified with GenePix 7.0 software (Axon Instrument). The data were annotated through the UniProt DB.

### Cell culture

Human gastric cancer cell lines were purchased from the Korean Cell Line Bank. The MKN-28 cancer cell line has been reported to be cross-contaminated with MKN-74 cells [20]. Therefore, we refer to MKN-28 cells as MKN-74 cells. All cell lines were grown in RPMI-1640 medium (WELGENE, Seoul, Korea) containing 10% (v/v) fetal bovine serum (Welgene) and 1% penicillin/streptomycin (Welgene) at 37 °C in a humidified, 5% CO2 atmosphere.

### Real-time quantitative RT-PCR

Total RNA was extracted using an RNeasy mini kit (Qiagen) according to the manufacturer’s instructions. cDNA was synthesized using a GoScript™ reverse transcription system (Promega, Madison, WI, USA). Quantitative real-time PCR was performed with SYBR (Promega) using a real-time PCR system (Applied Biosystems, Foster City, CA, USA). Primer information for the quantitative real-time PCR is provided in Supplementary Table S1.

### NINJ2 knockdown using a lentiviral particle and SiRNA

For stable NINJ2 knockdown in ECF-R MKN-74 cells, we used two shRNA constructs targeting different regions of the NINJ2 gene (isoform-1, isoform-2, and isoform-3) to produce TRCN0000063773 (clone 1), TRCN0000063775 (clone 2), and TRCN0000063776 (clone 3). The negative control used non-targeting pLKO.1-puro shRNA (SHC002). The pMDLg/pRRE, pRSV-Rev, and pMD2.G plasmids were a gift from Didier Trono (Addgene plasmids #12251, #12253, and #12259) [21]. The shRNA clones (TRCN0000063773, TRCN0000063775, and SHC002) were transfected into 293T cells with pMDLg/pRRE, pRSV-Rev, and pMD2.G, respectively, using Fugene HD (Promega) according to the manufacturer’s instructions. After 48 h, the supernatants were collected and filtered. Next, ECF-R MKN-74 cells were transduced with the lentiviral particles and selected in growth medium containing puromycin.

siRNA for transient NINJ2 (isoform-1, isoform-2, and isoform-3) knockdown were synthesized by GenePharma (China), and sequence information is provided in Supplementary Table S2. WT and ECF-R MKN-74 cells were incubated with 20 µM of siRNA, 7.5 µL of TransIT-X2 transfection reagent (Mirus Bio Corporation, Madison, WI, USA), and 250 µL of serum-free medium for 30 min at room temperature. The mixture was added to cells and incubated for 72 h. Transfection was performed every 3 days for 15 days.

### Generation of stable NINJ2 overexpressing MKN-74 cells

For *NINJ2*-overexpression in MKN-74 cells, NINJ2 isoform-1 (NP_057617.3) and isoform-2 (NP_001281275.1) were cloned into the pHTC HaloTag^®^ CMV-neo vector (Promega, G7711). The NINJ2 Iso-1 and Iso-2 vectors were transfected into MKN-74 cells using ViaFect™ transfection reagent (Promega) according to the manufacturer’s instructions. Transfected cells were selected in growth medium containing G-418 (Promega).

### Cell survival assay

Cell survival was assessed using CCK-8 as described above. Each cell line was seeded in a 96-well plate. After 24 h, an appropriate concentration of ECF was administered for 72 h. The cells were then subjected to the CCK-8 assay. The absorbance value of the mock control group was set as 100%, and then the percentage of surviving cells was calculated.

### Flow cytometry

For surface staining, cancer cells were collected in FACS buffer (Gibco, New York, NY, USA) containing 1% FBS (Welgene) and 0.1% bovine serum albumin (BSA) (MP Biomedicals, Irvine, CA, USA) and blocked using an Fc receptor blocker (Miltenyi Biotec, Bergisch Gladbach, Germany). Then, the cells were stained with antibodies specific for CD44-APC/cy7 (Biolegend, San Diego, CA, USA) and NINJ2 (R&D Systems, Minneapolis, MN, USA). To analyze the cell cycle, cancer cells were maintained in growth medium supplemented with 30 µM BrdU (Sigma-Aldrich) for 1 h. The BrdU-incorporated cells were fixed, permeabilized (Biolegend), and incubated with 300 µg/ml of DNase for 30 min at 37 °C. Then, the cells were stained with anti-BrdU APC antibody (Biolegend) and Hoechst 33342 (Thermo Fisher Scientific, Waltham, MA, USA). Appropriate isotype controls, fluorescence minus one controls, and secondary antibodies (Alexa Fluor 488) for NINJ2 staining were used. Flow cytometry analysis was performed using an LSR II (BD Biosciences, San Jose, CA, USA).

### Western blotting

Cell lysates for Western blot analysis were harvested using the RIPA buffer (Thermo Fisher Scientific). The antibodies were as follows: anti-NINJ2 antibody (AF5056) and anti-sheep IgG-HRP (HAF016) were purchased from R&D Systems; anti-CDC25a antibody (ab989), anti-CDK2 antibody (ab32147), anti-CDK4 antibody (ab108357), anti-CDK6 antibody (ab124821), anti-cyclin D1 antibody (ab134175), anti-cyclin E1 antibody (ab133266), anti-total Rb antibody (ab181616), anti-Rb (phospho Ser780) antibody (ab47763), anti-Rb (phospho Ser795) antibody (ab47474), anti-p27^KIP1^ antibody (ab32034), anti-mouse IgG-HRP antibody (ab6728), and anti-rabbit IgG-HRP antibody (ab6721) were purchased from Abcam; anti-GAPDH antibody (sc-47724) and anti-rat IgG-Texas Red antibody (sc-2782) were purchased from Santa Cruz; anti-phospho-VE-cadherin (Tyr731) (44-1145G), anti-rabbit IgG-Alexa Fluor 488 antibody (A11034), anti-sheep IgG-Alexa Fluor 555 antibody (A21436), and anti-mouse IgG-Alexa Fluor 488 antibody (A11001) were purchased from Life Technologies; anti-ATF-2 (35031T) was purchased from Cell Signaling Technology; and anti-phospho-ATF2 (Ser112 or Ser94) (CSB-PA163743), anti-phospho-VAV2 (Tyr142) (CSB-PA060057), anti-VAV2 (CSB-PA004442), anti-JUND (CSB-PA04469A0Rb), and anti-phospho-JUND (Ser255) (CSB-PA011303) were purchased from Cusabio.

### ECF sensitivity testing in gastric cancer organoids

Organoids were dissociated using TrypLE for homogeneous size. The dissociated single cells were seeded at 20,000 cells per well (48 well plates) with Matrigel, and organoid complete growth medium was added after incubation for 20 min at 37 °C. 7–10 days later, the organoids were collected from the Matrigel by the addition of Dispase II (1 mg/mL, 30 min at 37°C). Organoids were seeded at 250 organoids per well into a white 96-well plates. After one hour, ECF was added at the desired concentrations. Three days later, ATP levels were measured using a CellTiter-Glo 3D cell viability assay kit (Promega).

### Co-Immunoprecipitation

NINJ2 complex proteins in stable NINJ2-HaloTag MKN-74 cells were isolated using the HaloTag pull-down system (G6504, Promega) according to the manufacturer’s instructions. NINJ2-interacting proteins were identified via LC-MS/MS and Western blotting.

### Binding kinetics analysis using surface plasmon resonance

SPR experiments were performed in a Biacore 3000 (GE Healthcare, Madison, WI, USA). For immobilization, we used HBS-T (10 mM HEPES, 150 mM NaCl, 0.005% Tween20) pH 7.4 as a running buffer and flow rate was 10 µl/min. After activation using the 200 mM EDC and 100 mM NHS on the carboxymethyldextran chip (XanTec bioanalytics GmbH, CMD200M), extracellular domain (Met1-Thr65) of recombinant human NINJ2 (R&D systems) 50 µg/mL in 5 mM acetate buffer (pH 4.0) and reference were immobilized through amine coupling. To block, 1 M ethanolamine HCl for 7 min were injected. For kinetic evaluation, 5-concentration (231, 116, 58, 29 and 14 nM) of recombinant human Periostin (Sino Biological, China) were injected at 30 µL/min flow rate for a total of 2 min as an association phase. In the dissociation phase were carried out total of 5 min.

### Animal model

Five-week-old male BALB/c nu/nu mice were purchased from Orient Bio, Inc. (Sungnam, South Korea) and housed in a specific pathogen-free facility. The Institutional Animal Care and Use Committee approved the protocols for the animal study (No. 2023 − 0239), and all animals were maintained and used in accordance with the Guidelines for the Care and Use of Laboratory Animals of the Department of Laboratory Animal Resources, Yonsei Biomedical Research Institute, Yonsei University College of Medicine. To check whether the ECF-R cancer cells established in vitro had acquired resistance that would transfer to a xenograft model, 1.0 × 10^7^ ECF-R cancer cells or parental cancer cells were implanted subcutaneously in the flank area of each mouse. Two-dimensional tumor size was measured twice per week, and the tumor volume was calculated using Eq. (1):

Volume = ½ × a × b2 (1).

where a is the longest diameter, and b is the perpendicular diameter [22]. The mice were then euthanized, and the harvested tumors were paraffin-embedded after fixation using with 4% paraformaldehyde. To determine the anti-cancer effects of NINJ2 K/D on vulnerability to ECF, NINJ2 or control siRNA were synthesized by GenePharma. Sequence information is provided in Supplementary Table S2. When the tumors reached about 100 mm^3^, the ECF-R cancer bearing mice were randomly divided, and each mouse received intratumoral injections of NINJ2-targeting siRNA or control siRNA at the dose of 25 µg/mouse using in vivo-jetPEI (Polyplus-transfection) every 3 days. The mice also received intratumoral injections of 5.7 mg/kg of epirubicin, 6.67 mg/kg of cisplatin, and 22 mg/kg of 5-FU once a week.

### Immunohistochemistry staining

Formalin-fixed, paraffin-embedded tumor tissues were evaluated using anti-NINJ2, anti-CD44, and anti-cyclin D1 antibodies. Paraffin sections were deparaffinized in Histo-Clear (National Diagnostics, Atlanta, GA, USA) and rehydrated by gradually reducing the ethanol concentration from 100 to 95% and then to 90%. Antigen epitopes were then unmasked using Tris-EDTA buffer (pH 9.0). Subsequently, the slides were incubated at 4 °C with the primary antibodies. After overnight incubation, the slides were incubated with horseradish peroxidase-conjugated secondary antibodies, followed by incubation with diaminobenzidine (DAB) solution for 10 min.

### Statistical analysis

The results are presented as the mean ± SEM. Statistical comparisons between two different samples were made using the Mann-Whitney U test. To compare multiple samples, statistical significance was assessed using analysis of variance (ANOVA), and adjustment for multiple comparisons was done using Tukey’s test, the Tamhane test, or the Sidak test, as appropriate. Statistical significance was set at less than 0.05. Statistical tests were performed using SPSS version 25 (SPSS Inc, Chicago, IL, USA) and GraphPad Prism 10.

## Results

### NINJ2 expression is significantly elevated in ECF-R gastric cancer cells

**Fig. 1:**
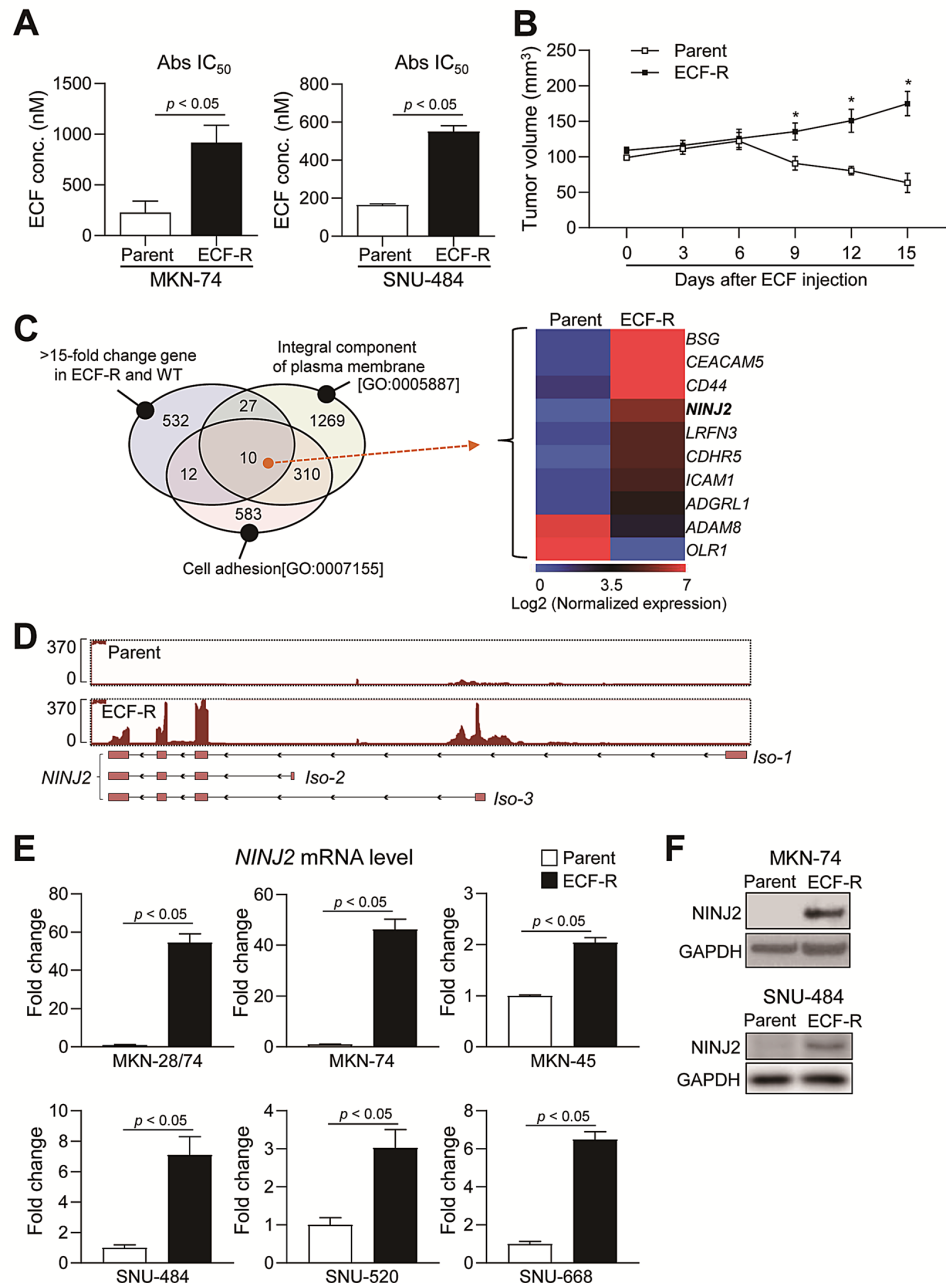
NINJ2 expression in ECF-R gastric cancer cells. (**A**) Representative IC_50_ values for parent (WT) and ECF-resistant (ECF-R) MKN-74 and SNU-484 cells. The IC_50_ values are given in an equation using a four-parameter logistic curve. (**B**) Parent and ECF-R MKN-74 cells were individually transplanted into nude mice and left until the tumor reached a volume of 100 mm^3^. The tumor-bearing mice were given ECF, and the tumor volume was measured. *p < 0.05 versus parent at each time point. (**C**) (Left) Venn diagram showing top genes with 15-fold changes in the RNA-sequencing of WT and ECF-R genes related to “Integral component of plasma membrane (GO:0005887)” and “Cell adhesion (GO:0007155).” (Right) Heatmap showing the gene expression level associated with resistance and the two gene ontology categories. (**D**) RGV analysis for the human NINJ2 isoform based on RNA-sequencing of WT and ECF-R (hg19 base) cells. (**E**) mRNA levels of *NINJ2* was analyzed by quantitative real-time PCR in parent and ECF-R cells from the MKN-28/74, MKN-74, MKN-45, SNU-484, SNU-520, and SNU-688 lines. (**F**) Protein levels of NINJ2 in parent and ECF-R MKN-74 and SNU-484 cells were determined by Western blot analysis. ECF, epirubicin, cisplatin, and 5-fluorouracil; ECF-R, ECF-resistance. Data are presented as mean ± SD

To establish ECF-R gastric cancer cells, we measured the IC_50_, IC_70_, and IC_80_ values of ECF in human gastric cancer cell lines according to the origin of the specimens (Supplementary Figure S1A). We then treated the MKN-74 (intestinal-type) and SNU-484 (diffuse-type) gastric cancer cell lines with gradually increasing doses of ECF corresponding to the IC_50_, IC_70_, and IC_80_ values. These cells were subjected to repeated drug-on (3 days) and drug-off (1–3 weeks) cycles for more than 2 months (Supplementary Figure S1B). To validate the establishment of ECF-R cells, we measured drug sensitivity in vitro and in vivo. We found that the IC_50_ values of ECF-R cells were markedly higher in intestinal- and diffuse-type gastric cancer cell lines compared to those in their parental cells (Fig. 1A, Supplementary Figure S1C). In the xenograft model, the volume of tumors derived from ECF-R cancer cells greatly increased even after ECF treatment, but decreased in the control groups (Fig. 1B).

To explore molecular changes associated with ECF resistance, we performed transcriptome analysis with bulk RNA-sequencing, identifying 581 genes that changed more than 15-fold between ECF-R and parental cancer cells (Fig. 1C). To identify membrane proteins that may be suitable targets for therapeutic agents, we analyzed integral components of the plasma membrane (Gene Ontology, GO:0005887). We narrowed the list to cell adhesion molecules (GO:0007155), such as CD44 and N-cadherin, which are key factors in tumorigenesis, chemoresistance, and cancer stem cell plasticity [23]. Finally, we selected 10 overlapping genes among the 581 genes using Gene Ontology analysis (Fig. 1C). We determined the protein levels of these ten overlapping molecules in normal tissues using the ProteomicsDB database (accessed on Oct 1st, 2024) [24, 25]. BSG, CD44, ICAM1, and CEACAM5 were ubiquitously expressed in various normal tissues, including the stomach (Supplementary Figure S1D). However, NINJ2 showed low expression levels in the frontal cortex, spinal cord, pancreas, and prostate of healthy humans (Supplementary Figure S1D-F). Therefore, we focused on NINJ2 as a novel biomarker of chemoresistance in gastric cancer.

Furthermore, NINJ2 isoform-3, but not isoforms 1 and 2, was mainly expressed in ECF-R gastric cancer cells (Fig. 1D). Notably, NINJ2 isoform-3 was rarely expressed in normal tissues (Supplementary Figure S1F). ECF-R primary human gastric cancer cell lines (SNU-488 and SNU-520) and metastatic human gastric cancer cell lines (MKN-28/74, MKN-74, MKN-45, and SNU-668) showed consistently and significantly increased *NINJ2* mRNA levels (Fig. 1E). Furthermore, MKN-74 and SNU-484 ECF-R human gastric cancer cells exhibited increased NINJ2 protein levels (Fig. 1F). These findings indicate that the expression of NINJ2 as a surface protein can be used as a novel biomarker for acquired ECF resistance in both intestinal and diffuse gastric cancer cells.

### CD44^+^ gastric cancer-initiating cells are enriched in a NINJ2-dependent manner

**Fig. 2:**
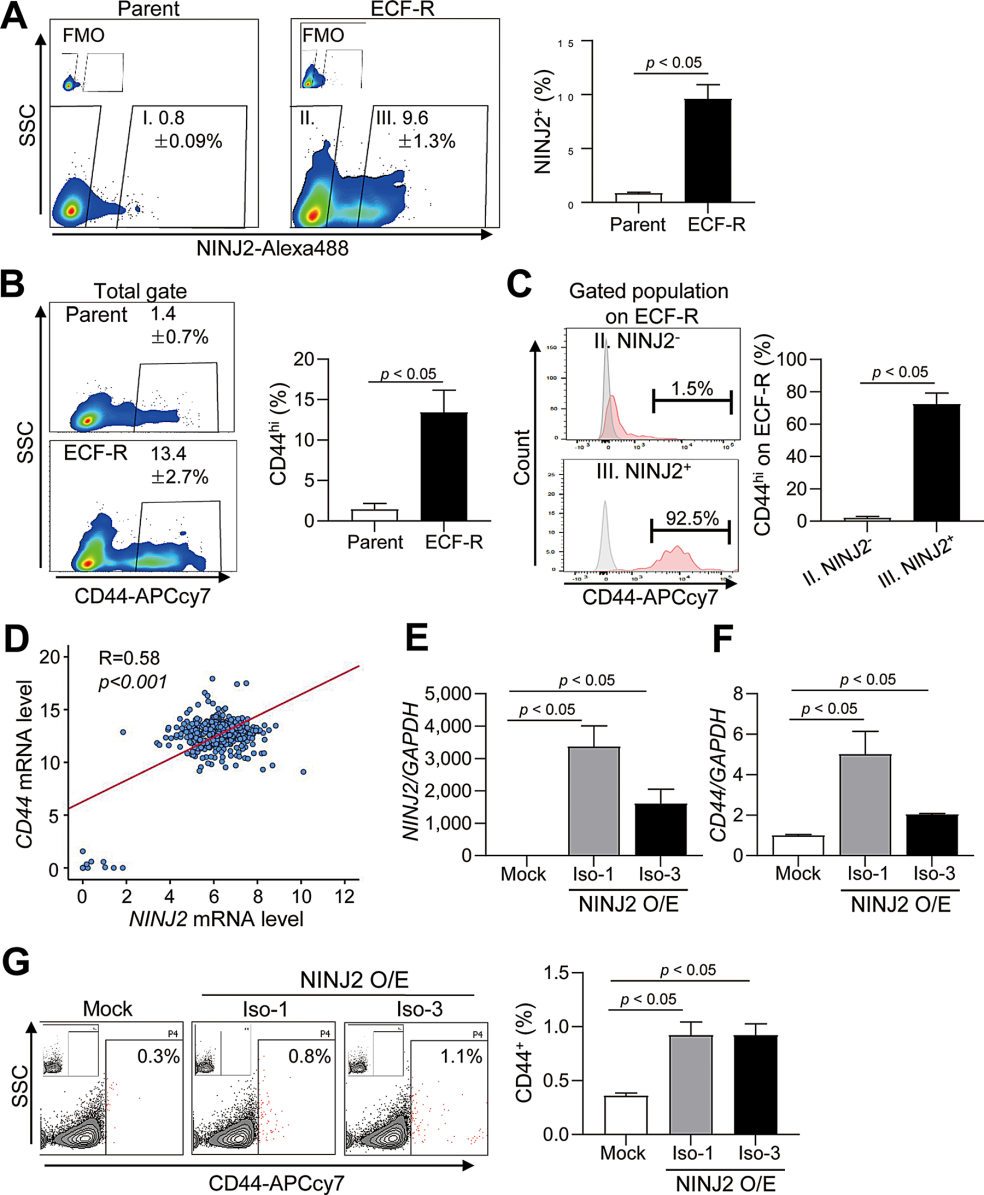
NINJ2 induces CD44 expression and cancer-initiating cells. (**A**) (Left) FACS plot showing the surface expression of NINJ2 on parent and ECF-R MKN-74 cancer cells. The I and III populations are NINJ2-expressing cells among parental and ECF-R cells, respectively. The II population is NINJ2^−^ cells among ECF-R cells. (Right) Quantification of NINJ2^+^ cells. (**B**) (Left) FACS plot showing surface expression of CD44 on parent and ECF-R MKN-74 cancer cells. (Right) Quantification of CD44^high^ cells. (**C**) (Left) FACS plot showing CD44 expression in gated NINJ2^−^ and NINJ2^+^ populations of ECF-R MKN-74 cancer cells. (Right) Quantification of the NINJ2^+^CD44^hi^ population. (**D**) NINJ2 and CD44 correlation analysis in human gastric adenocarcinoma (*n* = 426) from the The Cancer Genome Atlas (TCGA) and oncoSG dataset. (**E**) NINJ2 isoform-1 (NP_057617.3) and isoform-3 (NP_001281275.1) overexpression efficiency was analyzed by quantitative real-time PCR. (**F**) *CD44* mRNA level in NINJ2-overexpressing MKN-74 cells was measured by quantitative real-time PCR. (**G**) (Left) Percentage of CD44^high^ cells in stable mock-, NINJ2 isoform 1-, and NINJ2 isoform 3-overexpressing MKN-74 cells measured by FACS analysis. (Right) Quantification of the CD44^high^ cells. O/E, overexpressing. Data are presented as mean ± SD

CD44 expression in gastric cancer cells is associated with chemoresistance [26, 27]. Previous studies demonstrated that ablation of CD44 suppressed gastric tumor development in a transgenic mouse model of gastric cancer [28]. In this context, we confirmed that the proportion of NINJ2-positive cells in the ECF-R cancer cells (9.6 ± 1.3%) was higher than that in the parental cells (0.8 ± 0.09%) (Fig. 2A). Next, the proportion of CD44-expressing gastric cancer cells in ECF-R gastric cancer cells (13.4 ± 2.7%) was higher than that in the parental cells (1.4 ± 0.7%) (Fig. 2B). Similarly, we observed a significant increase in CIC markers, such as CD44, CD44v9, and CD24, in ECF-R gastric cancer cells compared with those in the parental cells (Supplementary Figure S2A). Furthermore, most NINJ2^+^ cells in the ECF-R gastric cancer cells also highly expressed CD44, and most of the NINJ2^−^ cells were also CD44^−^ cells (Fig. 2C, Supplementary Figure S2B). Furthermore, we generated tumor spheroids (enriched in CICs) to evaluate NINJ2 expression in spheroids. Tumor spheroids derived from MKN-74 gastric cancer cells showed significantly increased *NINJ2* mRNA levels (Supplementary Figure S2C). These spheroids also showed co-localized expression of NINJ2 and CD44, with the NINJ2^+^CD44^hi^ CICs located along the spheroid periphery (Supplementary Figure S2D). These findings indicate that the NINJ2^+^-enriched chemoresistant gastric cancer cells primarily consisted of CICs with high CD44 expression.

Next, to investigate the clinical correlation between NINJ2 and CD44 levels, we analyzed data from human gastric adenocarcinoma samples (*n* = 426) in The Cancer Genome Atlas (TCGA) and the oncoSG [29] dataset using cBioPortal [30, 31]. NINJ2 expression was significantly positively correlated with *CD44* mRNA levels (Fig. 2D). To determine whether CD44 expression was increased by NINJ2, we generated stable gastric cancer cells overexpressing (O/E) NINJ2 isoform-1 and -3 (Fig. 2E). These cells showed significant increases in both *CD44* mRNA expression and CD44^+^ CIC population (Fig. 2F-G). To determine the frequency at which gastric CICs exhibit tumorigenic properties, we tested tumor sphere formation using an in vitro limiting dilution assay. We found that NINJ2 isoform-1 and − 3 O/E MKN-74 cells were enriched in CICs (Supplementary Figure S2E). Similarly, tumor spheres formed in NINJ2 O/E MKN-74 cells were significantly more abundant than those formed in mock MKN-74 cells (Supplementary Figure S2F-G). Overall, these results indicate that CD44^+^ CICs were enriched in an NINJ2-dependent manner.

### NINJ2 increases chemoresistance and induces cell cycle arrest in gastric cancer cells

**Fig. 3:**
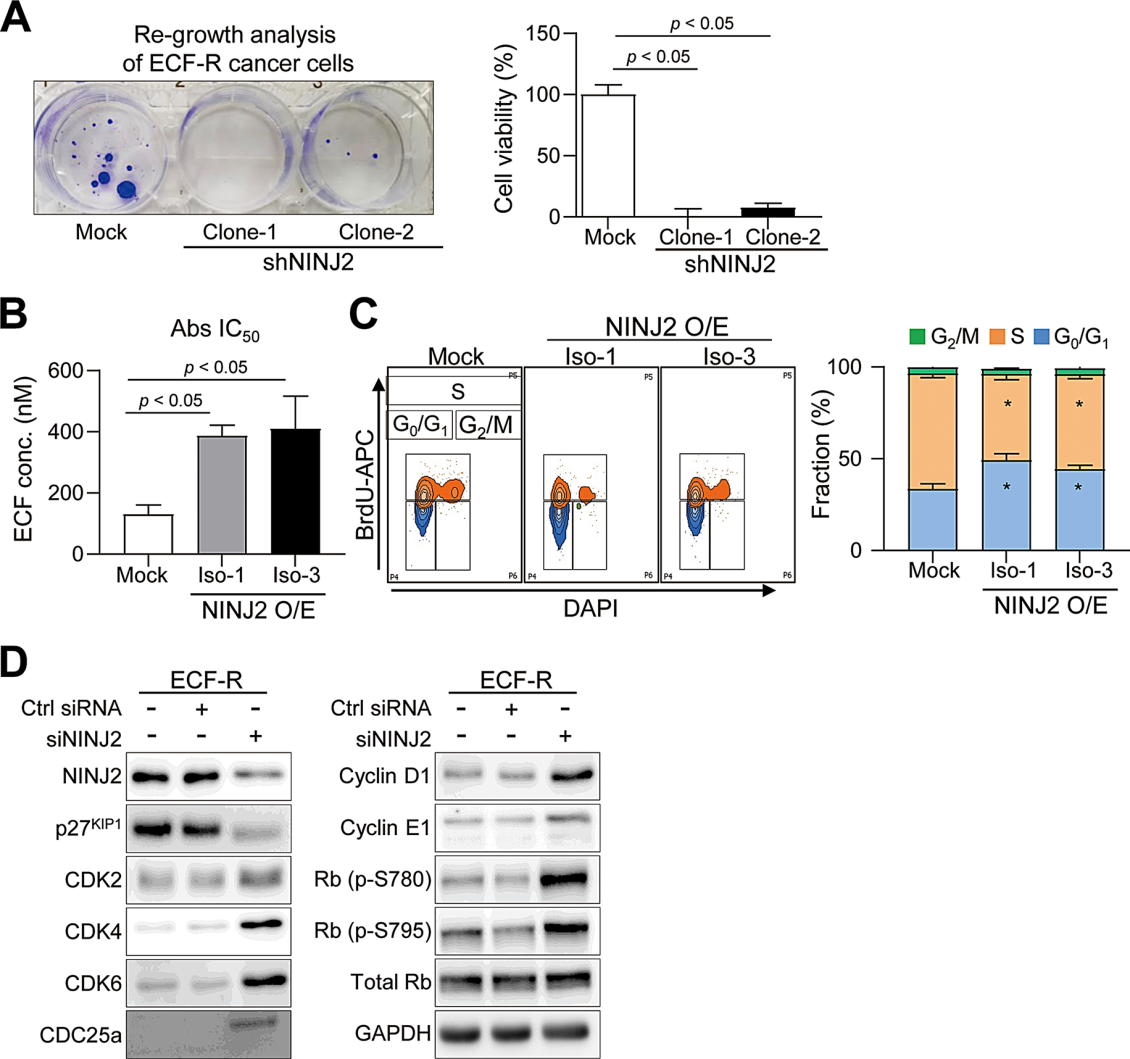
NINJ2 in ECF-R regulates chemo-sensitivity and cell cycle arrest. (**A**) ECF-R MKN-74 cells were transduced with shRNA lentiviral particles targeting human NINJ2 (clone 1 and clone 2) or the pLKO.1-puro empty control. Viability analysis using crystal violet staining (Left) and the CCK-8 assay (Right) 3 weeks after ECF treatment in mock and NINJ2 K/D ECF-R MKN-74 cells. (**B**) Representative ECF IC_50_ values for MKN-74 cells overexpressing NINJ2 isoform-1 and isoform-3. (**C**) (Left) Cell cycle status of mock-, NINJ2 isoform-1-, and isoform-3-O/E MKN-74 cells. (Right) Quantification of the left FACS plots. *p < 0.05 versus mock (S-phase reduction), #*p* < 0.05 versus mock (G0/G1-phase increase). (**D**) Western blot analysis of NINJ2, p27^KIP1^, CDK2, CDK4, CDK6, CDC25a, cyclin D1, cyclin E1, Rb (p-S780), Rb (p-S795), total Rb, and GAPDH in scramble siRNA and NINJ2 K/D MKN-74 cells. K/D, knockdown. Data are presented as mean ± SD

To evaluate whether NINJ2 is involved in chemoresistance, we generated stable NINJ2 knockdown (K/D) ECF-R gastric cancer cells. To analyze the changes in chemosensitivity caused by NINJ2 K/D, we selected the most highly effective shRNAs (clone 1 and clone 2) among the three clones to target NINJ2 isoform-1 and isoform-3 (Supplementary Figure S3A). Compared to mock ECF-R cells, stable NINJ2 K/D ECF-R cells prevented regrowth 3 weeks after ECF treatment (Fig. 3A). Consistent with this, NINJ2 K/D ECF-R cells established using siRNA showed decreased cell viability after ECF treatment (Supplementary Figure S3B). Conversely, NINJ2 isoform-1 and − 3 O/E significantly diminished the effectiveness of ECF treatment in gastric cancer cells (Fig. 3B, Supplementary Figure S3C).

It was previously reported that quiescent cancer cells are resistant to chemotherapies such as 5-FU and cisplatin, which target rapidly proliferating cells [32]. Therefore, we analyzed cell cycle changes associated with NINJ2 expression. Both NINJ2 isoform-1 and − 3 O/E gastric cancer cells showed increased cell cycle arrest, with inhibition of the G_0_/G_1_ phase to the S phase, resulting in anti-proliferative activity (Fig. 3C, Supplementary Figure S3D). As a cyclin-dependent kinase (CDK) inhibitor that inhibits proliferation, the p27^KIP1^ protein was downregulated by NINJ2 K/D. Additionally, the phosphorylation of CDK2, CDK4, CDK6, CDC25a, cyclin D1, cyclin E1, and retinoblastoma, which are key factors for the progression from the G_0_/G_1_ phase to the S phase, was increased by NINJ2 K/D (Fig. 3D).

Meanwhile, to evaluate the association between chemoresistance and invasive potential, we performed a gelatin-coated transwell invasion assay. ECF-R cells exhibited significantly increased invasive potential compared to parental cells (Supplementary Fig. S3E). Furthermore, analysis of EMT marker expression revealed that vimentin levels were significantly higher in ECF-R cells than in parental cells (Supplementary Fig. S3F). Notably, siNINJ2 treatment significantly reduced vimentin expression in ECF-R cells (Supplementary Fig. S3F). Finally, knockdown of NINJ2 significantly decreased the invasive potential of ECF-R cells, consistent with the reduction in vimentin levels (Supplementary Fig. S3G).

Collectively, these findings demonstrate that elevated NINJ2 expression in ECF-R gastric cancer cells drives chemoresistance by inducing cell cycle arrest and promoting invasiveness.

### Periostin is the NINJ2 binding protein responsible for the chemoresistance induced through VAV2 activation

**Fig. 4:**
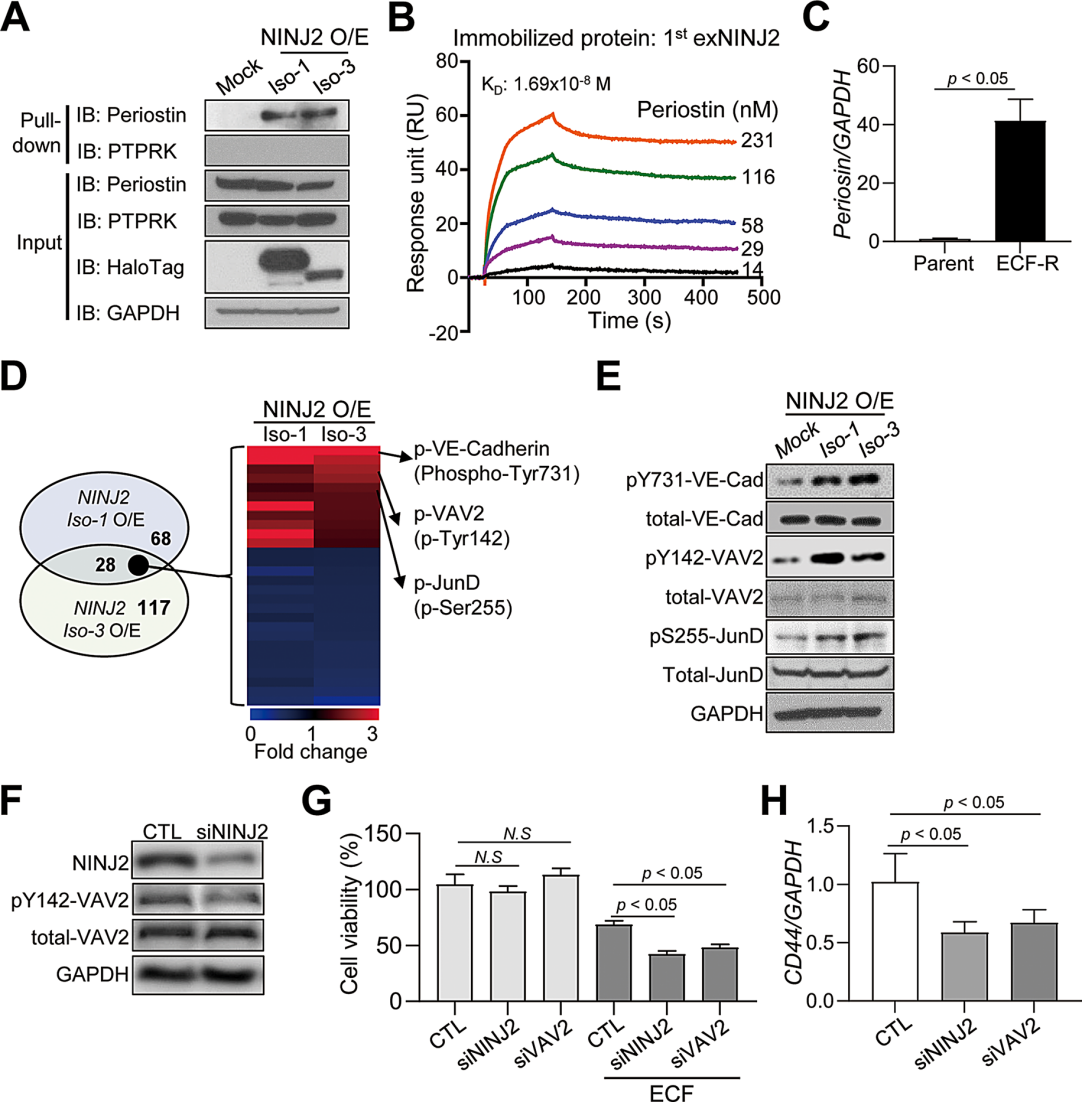
NINJ2 activates VAV2 signaling by interacting with periostin. (**A**) NINJ2/periostin interaction from stable NINJ2-HaloTag MKN-74 cancer cells was confirmed by co-immunoprecipitation. (**B**) Kinetics for NINJ2/periostin interaction was estimated by SPR analysis. The extracellular domain of NINJ2 immobilized through amine coupling and periostin at the five concentration (231, 116, 58, 29 and 14 nM) were flowed. (**C**) *periostin* mRNA levels in parent or ECF-R MKN-74 cancer cells. (**D**) Signaling analysis from NINJ2-overexpressing MKN-74 cells analyzed through site-specific and phospho-specific antibodies. (**E**) Western blot analysis of VE-cadherin, VAV2, and JunD in mock or NINJ2-overexpressing MKN-74 cells. (**F**) ECF-R MKN-74 cells were treated with *NINJ2* siRNA or control siRNA to analyze the expression of p-VAV2/VAV2 by western blotting. (**G**) ECF-R MKN-74 cells with *NINJ2* or scramble siRNA knockdown were evaluated for cell viability using CCK-8 assay after ECF (IC_50_) treatment. (**H**) Expression of *CD44* mRNA level in *NINJ2* or scramble siRNA-treated ECF-R MKN-74 cells. Data are presented as mean ± SD

To identify proteins that interact with NINJ2, we performed a pull-down assay of NINJ2 complex proteins and liquid chromatography–tandem mass spectrometry (LC-MS/MS). The process is summarized in Supplementary Figure S4A. We identified periostin, protein tyrosine phosphatase receptor type K (PTPRK), RNA-binding protein 28, and fibrinogen gamma chain as potential NINJ2-interacting proteins. Periostin and PTPRK have previously been reported to be associated with drug resistance in cancer and cancer stem cells [33, 34]. We confirmed the specific interaction between periostin and NINJ2 using pull-down analysis followed by immunoblotting; however, we did not observe any interaction with PTPRK (Fig. 4A). To investigate the direct interaction between periostin and the NINJ2 extracellular domain, we performed surface plasmon resonance (SPR) analysis. The extracellular domain (Met1-Thr65) of recombinant human NINJ2 on the carboxymethyl dextran chip was immobilized by amine coupling. Five different concentrations of recombinant human periostin were injected onto the surface of the chips after stabilization and regeneration. Periostin interacted directly with the extracellular domain of NINJ2, and the calculated K_D_ was 1.69 × 10^− 8^ M (Fig. 4B). Periostin mRNA levels were significantly increased in ECF-R cancer cells (Fig. 4C). To investigate the mechanisms through which NINJ2 induces drug resistance, we performed a phospho-antibody array using NINJ2 O/E gastric cancer cells. NINJ2 O/E upregulated the expression of four phosphorylated proteins, VE-cadherin (phospho-Tyr731), VAV2 (phospho-Tyr142), and JunD (phospho-Ser255), by more than 1.5-fold (Fig. 4D, Supplementary Figure S4B), as confirmed by western blotting (Fig. 4E).

Periostin, which binds to NINJ2, is known to induce intracellular signaling by interacting with integrins such as αvβ3, αvβ5, α6β4, αMβ2, and αv [35–37]. Additionally, VAV2 tyrosine phosphorylation is activated upon ligand binding to αMβ2 and αv integrins in various cells [38, 39]. Therefore, we focused on VAV2 signaling in subsequent experiments. We used siRNA to specifically target NINJ2 in gastric cancer cells and investigated its role in chemoresistance. K/D of NINJ2 led to a significant decrease in VAV2 phosphorylation in ECF-R gastric cancer cells (Fig. 4F), which further reduced cell viability after ECF treatment. Notably, VAV2 K/D exhibited the same effect as NINJ2 K/D (Fig. 4G). NINJ2 or VAV2 K/D significantly decreased *CD44v9* mRNA levels, which is recognized as a CIC marker (Fig. 4H). These findings suggest that the NINJ2/periostin/VAV2 signaling axis plays a crucial role in mediating chemoresistance and highlight the potential of targeting this pathway to enhance the response to ECF therapy in gastric cancer.

### Inhibition of NINJ2 significantly enhances chemosensitivity in ECF-R cancer cells using a xenograft model

**Fig. 5:**
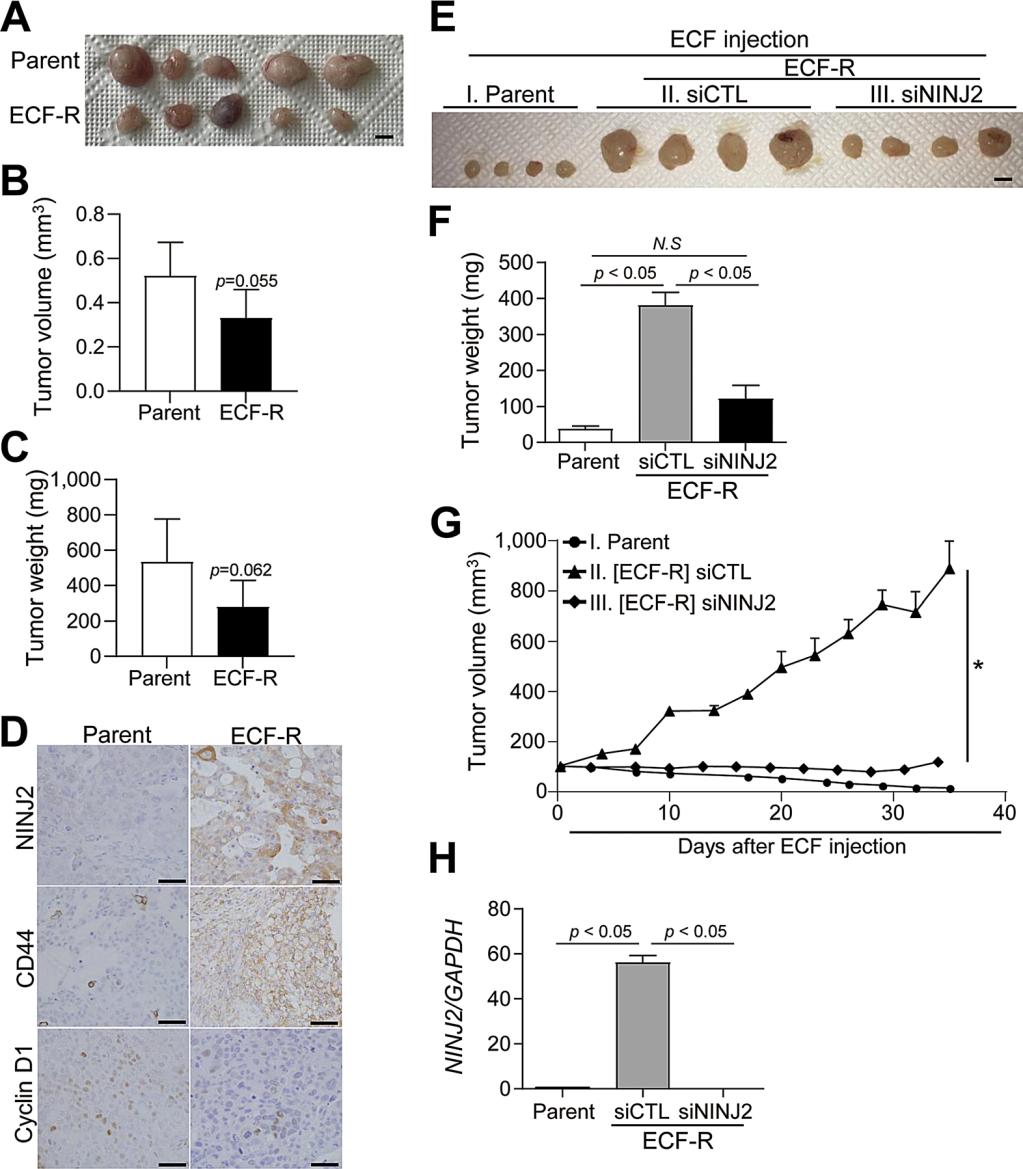
Role of NINJ2 in chemoresistant relapse of gastric cancer. (**A**) Parent or ECF-R MKN-74 cells were subcutaneously injected into nude mice and tumor growth was observed for 30 days. Scale bar, 5 mm. (**B**) Tumor volume, (**C**) and tumor weight. (**D**) Representative images of IHC for NINJ2, CD44, and Cyclin D1 in tumors from each group. Scale bar, 50 μm. (**E**-**G**) Parent MKN-74, *NINJ2* or scrambled siRNA-treated ECF-R MKN-74 cells were subcutaneously injected into nude mice treated weekly with ECF. (**E**) Representative tumor images. Scale bar, 5 mm. (**F**) Tumor weight and (**G**) tumor volume. (**H**) *NINJ2* mRNA level was measured with quantitative real-time PCR in tumors. Data are presented as mean ± SD

An ectopic tumor model was used to compare tumor growth rates between parental cells and ECF-R cells in vivo. Ectopic tumor growth was measured four weeks after cell injection (Fig. 5A). The mean volume and weight of tumors collected from the parent cell group were slightly greater than those from the ECF-R cell group (Fig. 5B-C), which was consistent with in vitro results. Moreover, cell cycle-related proteins were downregulated in the ECF-R cells compared to the parent cells. Furthermore, NINJ2^+^ and CD44^+^ cell proportions were increased in the ECF-R cell group compared to the parent cell group (Fig. 5D), as confirmed by immunohistochemistry (IHC). Conversely, Cyclin D1^+^ cells decreased in the ECF-R cell group compared to the parent cell group (Fig. 5D). Tumor RNA data supported these histological findings (Supplementary Figure S5A).

Next, we evaluated whether NINJ2 inhibition through multiple injections of polyvalent siRNA enhanced chemotherapeutic response in an ectopic tumor model. Consistent with in vitro findings, NINJ2 K/D markedly reduced in tumor growth upon ECF exposure in ECF-R cancer cells (Fig. 5E-G). Moreover, siRNA effectively downregulated NINJ2 expression in tumor tissues (Fig. 5H). These results indicate that NINJ2 plays a critical role in ECF resistance and that targeting NINJ2 offers a potential therapeutic strategy to overcome ECF resistance.

### NINJ2 expression is induced in ECF-R tumor organoids and shows clinical significance in disease progression

**Fig. 6:**
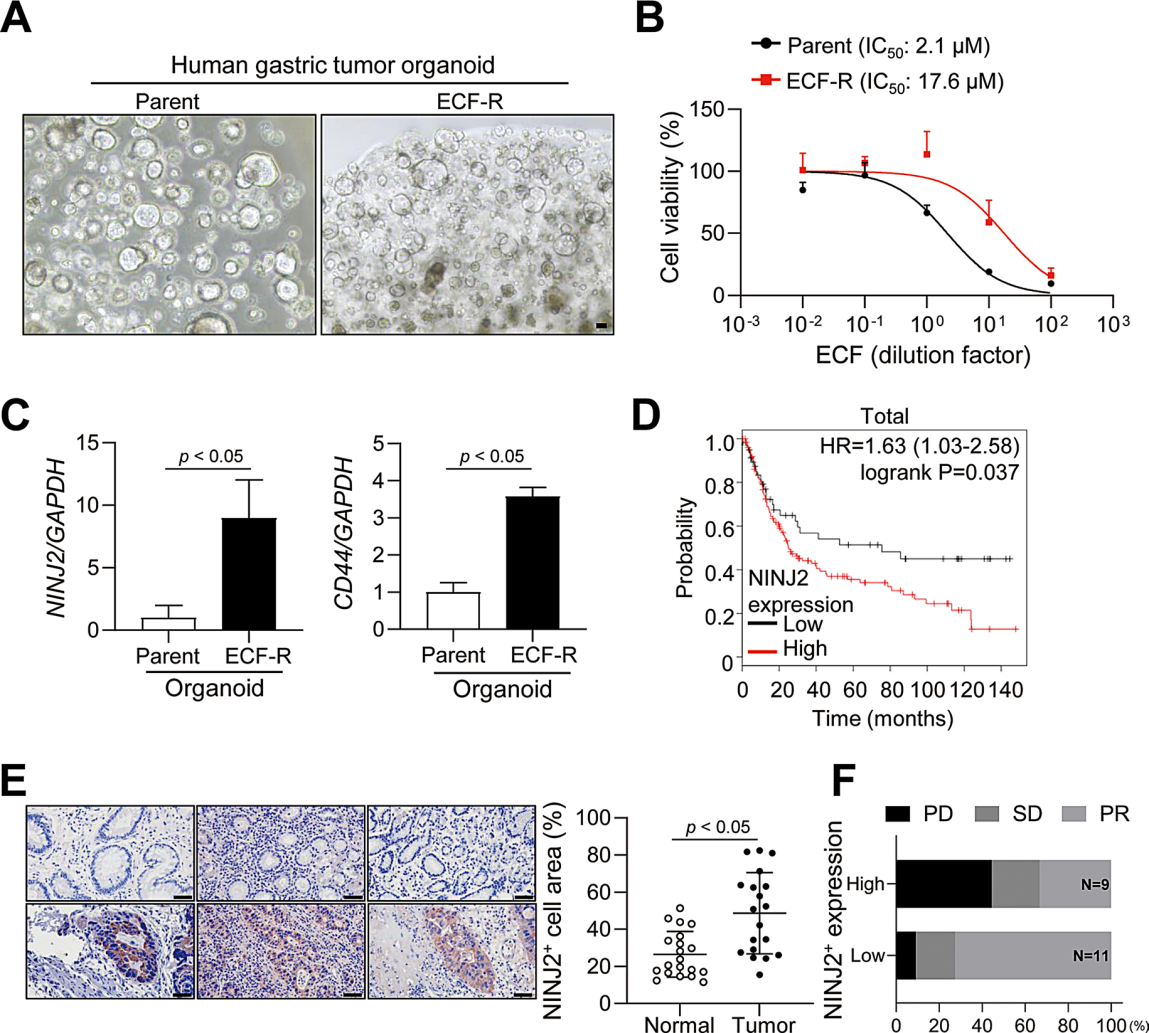
NINJ2 is upregulated in ECF-R tumor organoids and shows clinical importance. (**A**) Representative images of parental and ECF-R human gastric tumor organoids. Scale bar, 40 μm. (**B**) ECF IC_50_ values of parental and ECF-R tumor organoids. (**C**) *NINJ2* and *CD44* mRNA levels in parental and ECF-R human gastric tumor organoids (**D**) Kaplan–Meier curves for overall survival of gastric cancer patients in a public dataset (GSE15459). (**E**) Expression of NINJ2 in tumor tissues and their corresponding normal tissues. Scale bar, 50 μm. (**F**) Chemotherapy response variability of patients according to NINJ2 expression in gastric cancer tissues, stratified into low and high expression groups. Data are presented as mean ± SD

Tumor organoids from patients with gastric, pancreatic, and colorectal cancers provide a new platform for drug testing [40, 41]. First, we measured the IC_50_, IC_70,_ and IC_80_ values of ECF in patient-derived gastric tumor organoids (Supplementary Figure S6A). Using a method similar to that used to establish our ECF-R cells, we established ECF-R gastric tumor organoids (Fig. 6A-B). Consistent with our results in cancer cells, the ECF-R tumor organoids had increased *NINJ2* and *CD44* mRNA levels compared with the parental tumor organoids (Fig. 6C). Next, we analyzed the clinical relevance of NINJ2 expression to patient survival using Kaplan–Meier analysis and log-rank testing with public datasets (GSE15459) of patients with gastric cancer. Patients with high NINJ2 expression had poor survival (Fig. 6D). To validate the clinical relevance of NINJ2 expression in patients with gastric cancer, we performed IHC staining for NINJ2 in human gastric cancer tissues and corresponding normal tissues. Figure 6E shows that the NINJ2^+^ cell area was significantly upregulated in tumor tissues compared to that in normal gastric tissues. Furthermore, the analysis of patient tumors stratified by high and low NINJ2 expression revealed a correlation with disease progression. Patients with high NINJ2 expression exhibited a higher frequency of progressive disease and stable disease compared to the low NINJ2 expression group following surgery (Fig. 6F).

## Discussion

Chemoresistance remains a major obstacle in cancer treatment, especially in gastric cancer, where rapid resistance after first- and second-line therapies significantly contributes to poor survival. Therefore, developing new strategies to prevent chemoresistance could provide significant survival benefits for patients with gastric cancer. We established ECF-R gastric cancer cells and patient-derived tumor organoids through stepwise selection of various ECF doses. We identified NINJ2 as an activator of chemoresistance. NINJ2-expressing ECF-R cells highly expressed CD44, which is a gastric cancer stem cell marker. NINJ2 O/E in cancer cells increased the CD44^+^ cancer stem cell population and induced cell cycle arrest. NINJ2 K/D in ECF-R cancer cells enhanced chemosensitivity *in vitro* and *in vivo*. These findings suggest that NINJ2 could be a new therapeutic target to prevent the development of ECF tolerance in gastric cancer.

NINJ2 is typically expressed in mature sensory and enteric neurons and is associated with an increased risk of ischemic stroke [12]. Although previous studies have demonstrated that NINJ2 is upregulated in gliomas and colorectal cancer, the underlying mechanisms remain poorly understood [15, 16]. Here, we used LC-MS/MS, SPR, and co-immunoprecipitation to demonstrate that periostin is a NINJ2 binding partner in gastric cancer cells. Periostin is highly expressed in most solid tumors, such as stomach, pancreas, lung, breast, colon, and liver carcinomas [42, 43]. Furthermore, periostin levels are elevated in primary gastric cancer and metastatic lymph nodes when compared to benign gastric conditions [44]. Periostin promotes chemoresistance to cisplatin and 5-FU in gastric cancer cells through the Akt/p53 pathway [44] and enhances gemcitabine resistance in pancreatic cancer through the Akt/ERK pathway [45]. Additionally, periostin maintains cancer stem cells via Wnt signaling, and blocking it prevents metastasis [35]. These findings indicate functional overlap between NINJ2 and periostin. In this study, we found that the extracellular domain of NINJ2 strongly interacts with periostin. NINJ2 is a periostin receptor that plays an important role in chemoresistance. To define the molecular mechanism by which NINJ2 regulates ECF chemoresistance, we investigated the phosphorylation profile of NINJ2 using 1,318 site-specific antibodies. We demonstrated that the NINJ2 downstream pathway is linked to the VAV2 pathway. VAV2 has been reported to play a pivotal role in drug resistance, invasion, metastasis, and maintenance of cancer stem cells [46]. Based on our data and previously published data, we suggest that the NINJ2/periostin axis induces chemoresistance and CIC maintenance via VAV2 signaling. To further understand how NINJ2 promotes tumor aggressiveness beyond chemoresistance, we investigated its potential involvement in mesenchymal transition. Our findings demonstrate that increased NINJ2 expression in chemoresistant gastric cancer cells leads to upregulation of CD44, which in turn promotes vimentin expression and enhances invasive and mesenchymal characteristics. Previous studies have reported that CD44 directly induces vimentin gene transcription via EMT transcription factors like Slug, thereby facilitating EMT and metastatic progression in gastric cancer [47]. Also, high CD44 expression is known to be positively correlated with vimentin levels and EMT features in various cancers, including gastric and colon cancers [48]. Taken together, our findings suggest that NINJ2 drives tumor aggressiveness and represents a promising therapeutic target for inhibiting both drug resistance and metastasis in gastric cancer.

Targeting antigens in tumors is a powerful therapeutic strategy. For example, CAR-T cells targeting B-cell lymphomas can induce successful responses [49]. Similarly, T cells specific to the carcinoembryonic antigen (CEA) in colon cancer have shown positive responses [50]. However, CD19 and CEA are also expressed in normal polyclonal CD19^+^ B-lineage cells and colonic mucosa. Thus, the number of drugs that can be systemically administered is limited by their toxicity to normal cells. Antigens that are selectively expressed on cancer or chemoresistant cancer cells (but not on normal cells) could play an important role in targeting cancer cells. We found that NINJ2 is rarely expressed in normal tissues, but has a pivotal role in conferring chemoresistance in gastric cancer cells. Thus, NINJ2 antigens can be developed into specific targeted therapies for chemoresistant gastric cancer cells using siRNA with lipid nanoparticles, antibodies, peptide-pulse immune cells, or small molecules.

Since CD44 and CD133 have also been identified as markers of gastric cancer stem cells, we investigated how the levels of both proteins correlated with NINJ2 expression. In a xenograft model, we demonstrated that CD44 levels increased with NINJ2 expression, but CD133 levels did not change (data not shown). We hypothesized that chemoresistance in gastric cancer cells could be synergistically suppressed by the NINJ2/CD133 dual knockdown. However, NINJ2/CD133 dual inhibition did not confer a synergistic benefit compared with siNINJ2 alone in our xenograft model (data not shown). This suggests that the manipulation of NINJ2 activity may indirectly influence a CD133-related pathway or ligand, thereby negating the need for direct CD133 targeting.

The optimization of chemotherapy regimens for solid tumors, including gastric cancer, remains an ongoing area of investigation. Considering the mechanisms of resistance to combination chemotherapy regimens, such as ECF, which target DNA damage and synthesis, it is expected that analogous resistance mechanisms may operate in other combination therapies.

## Conclusions

Our findings reveal that NINJ2, a surface protein, is highly expressed in ECF-R gastric cancer cells and patient-derived tumor organoids. Inhibiting NINJ2 expression increased chemosensitivity both in vitro and in vivo. Moreover, we identified periostin as a NINJ2-interacting protein, and NINJ2 overexpression induced VAV2 activation, leading to drug resistance. In patients with gastric cancer, NINJ2 expression is significantly elevated in those with progressive disease, and high NINJ2 expression correlates with a poor survival rate. Hence, NINJ2 represents a novel regulator of chemoresistance and shows excellent potential as a therapeutic target for overcoming chemoresistance in gastric cancer.

## Electronic supplementary material

Below is the link to the electronic supplementary material.

Supplementary Material 1

## Data Availability

No datasets were generated or analysed during the current study.

## Abbreviations

CDK: Cyclin-dependent kinase
CEA: Carcinoembryonic antigen
CIC: cancer-initiating cell
ECF: Epirubicin, cisplatin, and 5-fluorouracil
ECF: R-ECF-resistant
IHC: Immunohistochemistry
K/D: Knockdown
LC: MS/MS-Liquid chromatography-tandem mass spectrometry
NCCN: National Comprehensive Cancer Network
NINJ2: Nerve injury-induced protein 2
O/E: Overexpressing
PTPRK: Protein tyrosine phosphatase receptor type K
SPR: Surface plasmon resonance
TCGA: The Cancer Genome Atlas
