# Supplementary material for: Targeted inhibition of Ninjurin2 promotes chemosensitivity in chemoresistant gastric cancer by suppressing cancer-initiating cells
Source: Biomark Res. 2025 Jun 15;13:84. doi: 10.1186/s40364-025-00792-0 (PMC12168268; doi:10.1186/s40364-025-00792-0)
Supplement: Supplementary file 1 — Supplementary Material 1 [file 40364_2025_792_MOESM1_ESM.docx]

Supplementary Methods and Materials

**IC50 calculation**The efficacy of a combination of epirubicin, cisplatin, and 5-FU was evaluated using 10-fold serial dilutions in gastric cancer cell lines, which were seeded onto 96-well plates 24 h earlier. Cell viability was assessed using CCK-8 assay (Sigma-Aldrich) after 3 days of treatment. The IC50 values were calculated using GraphPad Prism 10 (GraphPad Software, San Diego, CA, USA).

**Tumor sphere assay and limiting dilution assay**MKN-74 gastric cancer cells overexpressing NINJ2 isoform 1 or NINJ2 isoform 3 were seeded at 1,000 cells per well in a low-attachment 24-well plate (Corning, New York, NY, USA) in DMEM-F12 supplemented with 20 ng/ml of recombinant human-epidermal growth factor (rhEGF), 20 ng/ml of recombinant human basic fibroblast growth factor (rhbFGF), and 5 µg/ml of insulin. After 10 days, the number of tumorspheres was counted and analyzed using ImageJ. We also used quantitative real-time PCR and immunofluorescence staining to measure the mRNA and protein levels in tumorspheres derived from MKN-74 cells.

For the limiting dilution assay, mock-, NINJ2 isoform 1–, and NINJ2 isoform 3–overexpressing MKN-74 gastric cancer cells were plated at a range of cell concentrations (8–1,000 cells per well) through 2-fold serial dilutions in low-attachment 96-well plates (Corning). The plated cells were maintained in DMEM-F12 supplemented with 20 ng/ml of rhEGF, 20 ng/ml of rhbFGF and 5 of µg/ml insulin. The data were analyzed using the extreme limiting dilution assay.

**Immunofluorescence staining**Cancer cells and tumorspheres were fixed with 1% paraformaldehyde for 10 min and washed at least three times with phosphate-buffered saline (PBS). To stain frozen tissue sections, we subjected them to immunofluorescence staining after drying them at room temperature for 20 min. All samples were blocked with 1% BSA at room temperature for 30 min. The samples were incubated with primary antibodies followed by fluorescence-conjugated secondary antibodies. After 3 washes, mount solution containing 4,6-diamidino-2-phenylindole (Invitrogen, San Diego, CA, USA) was dropped onto the samples, and they were stored at 4°C in the dark. Fluorescence images were obtained using a Zeiss LSM700 laser scanning confocal microscope (Carl Zeiss Meditec, Jena, Germany).

**Organoid culture and the generation of ECF-resistant tumor organoids**Patient-derived human gastric tumor organoids (HCM-BROD-0115-C16, PDM-135) were purchased from ATCC. The organoids were cultured in Ad-DMEM/F12 supplemented with GlutaMAX (Invitrogen), penicillin/streptomycin (Welgene), B27 (Thermo Fisher Scientific), N-acetyl-L-cysteine (Sigma-Aldrich), Wnt3a-conditioned medium, RSPO1-conditioned medium (R&D Systems), recombinant noggin protein (PeproTech, Rocky Hill, NJ, USA), recombinant epidermal growth factor protein (PeproTech), gastrin (Sigma-Aldrich), recombinant fibroblast growth factor 10 protein (PeproTech), nicotinamide (Sigma-Aldrich), and A83-01 (Tocris, Bristol, UK).

To establish ECF-R tumor organoids, the organoids were treated with the IC50 concentration of ECF. After 72 h, the medium was changed to drug-free medium, and sub-culturing was performed twice. Next, the organoids were exposed to the appropriate IC70 and IC80 concentrations, and the above procedures were repeated. To prevent reversion to the chemo-sensitive equilibrium, the organoids were treated with the appropriate IC80 concentration of ECF every 3 weeks. All experiments were performed after sub-culturing. The ECF resistance of the organoids was validated in vitro.

**Cell invasion assay**

Cell invasion was assessed using a transwell system (Corning, NY, USA). An 8 µm pore-size polycarbonate membrane was coated with 0.2% gelatin solution for 1 h and then dried overnight. The upper chamber contained 1 × 10^5^ cells in a 24-well plate, while the lower chamber was filled with 650 µL media. After 24 h, the non-migrating cells were swabbed with a cotton-tipped applicator, fixed in methanol for 30 min, and stained with 1% crystal violet for 30 min.

**Western blotting**

Cell lysates for Western blot analysis were harvested using the RIPA buffer (Thermo Fisher Scientific). The antibodies were as follows: anti-vimentin antibody (MAB2105), anti-N-cadherin (AF6426) and anti-E-cadherin antibody (AF748) were purchased from R&D Systems.

**Table S1.**

Primer sequences for qRT-PCR (Human)

| **Primer** | **Sequence** |
| --- | --- |
| ***NINJ2*** | Forward 5′-GACCATCCTCTCACTACTACACC-3′ |
|  | Reverse 5′-GTAATGAAAACATTGATGACCACAGTG-3′ |
| ***CD44*** | Forward 5′-GGGCAGAAGAAAAAGCTAGTGA |
|  | Reverse 5′-AATGCACCATTTCCTGAGACTT |
| ***Periostin*** | Forward 5′-TCCTAGAAAGGATCATGGGAGAC |
|  | Reverse 5′-GTCACCGTCACATCCTATCTCA |
| ***CD44v9*** | Forward 5′-TTCTCTACATCACATGAAGGC |
|  | Reverse 5′-GCTTGATGTCAGAGTAGAAGT |
| ***CD24*** | Forward 5′-CCAAGCATCCTGAGCAACTC |
|  | Reverse 5′-TGATCTGAGATCGCACCACT |
| ***GAPDH*** | Forward 5’-TTGTCAAGCTCATTTCCTGGTATG-3 |
|  | Reverse 5’-TCTCTCTTCCTCTTGTGCTCTTG-3 |

**Table S2.**
siRNA sequences for *NINJ2* and control

| **Primer** | **Direction** | **Sequence** |
| --- | --- | --- |
| ***NINJ2*** | Sense | 5′-rGrUrArArGrGrCrArUrGrUrCrUrGrUrCrUrArArGrGrCrCd(AA)-3′ |
|  | Anti-sense | 5′-rGrGrCrCrUrUrArGrArCrArGrArCrArUrGrCrCrUrUrArCd(AA) -3′ |
| ***Control*** | Sense | 5′-UUCUCCGAACGUGUCACGUTT-3’ |
|  | Anti-sense | 5′-ACGUGACACGUUCGGAGAATT-3’ |

**Figure S1**


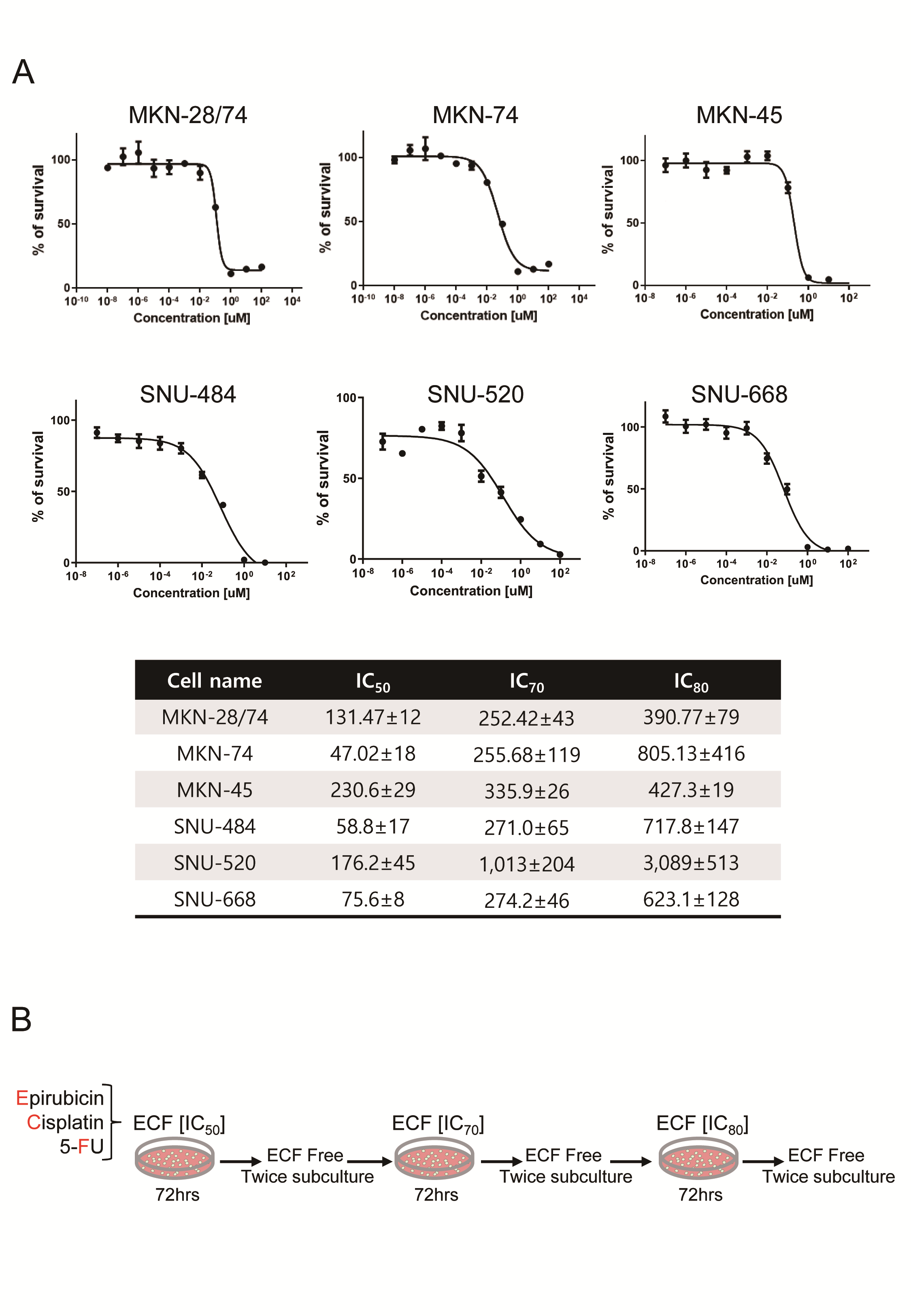

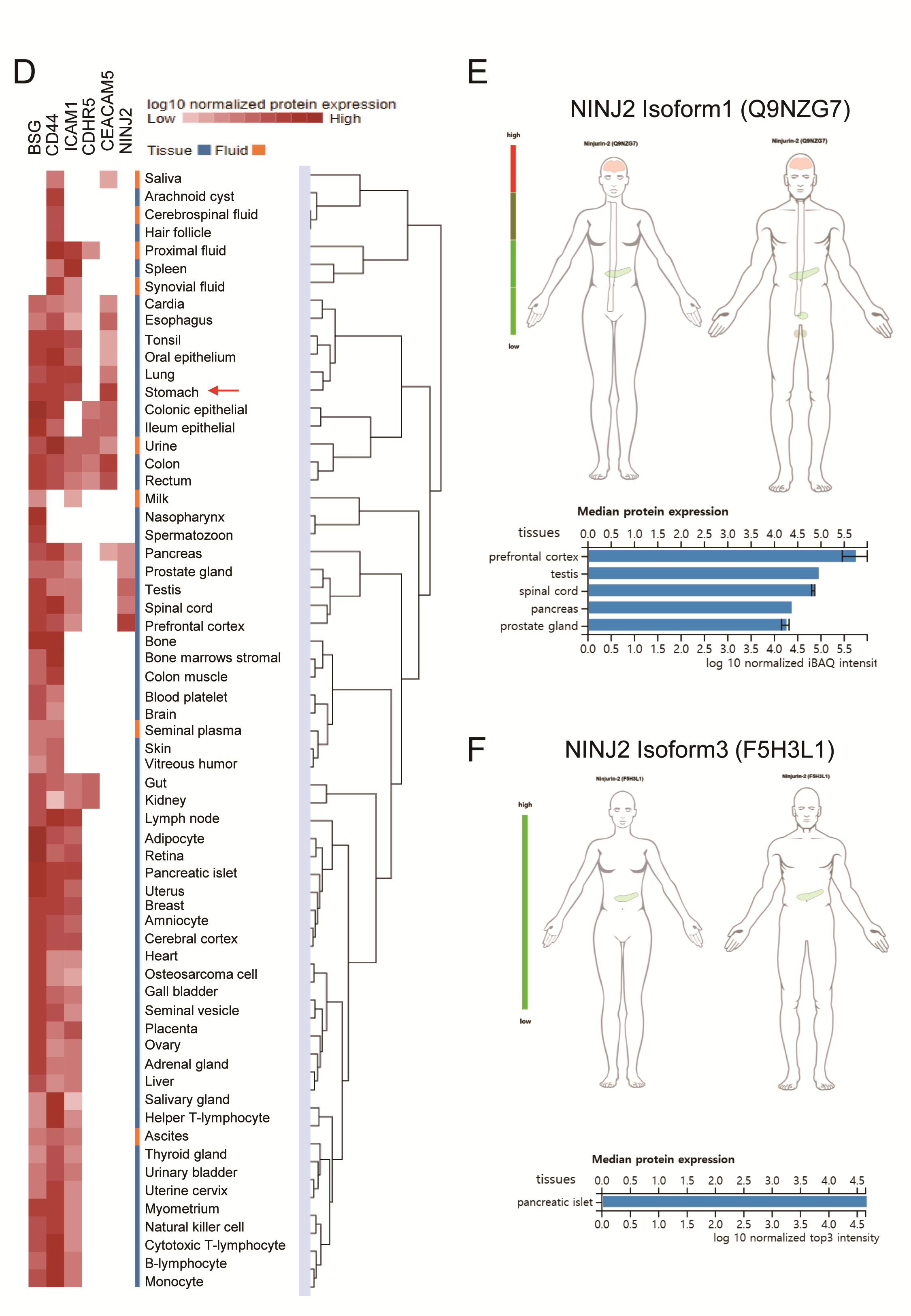


Figure S1. (A) (Top) Representative figures showing the IC_50_ values for MKN-28/74, MKN-74, MKN-45, SNU-484, SNU-520, and SNU-668 cells. (Bottom) Table of IC_50_, IC_70_, and IC_80_ values for MKN-28/74, MKN-74, MKN-45, SNU-520, SNU-484, and SNU-688 cells. IC_50_, IC_70_, and IC_80_ values given using four-parameter logistic curve equation. (B) Schematic figure for the experimental procedure. (C) (Top) Representative figures for the IC_50_ values of parent (WT) and ECF-resistant (ECF-R) MKN-28/74, MKN-74, MKN-45, SNU-484, SNU-520, and SNU-668 cells. (Bottom) Table of IC_50_, IC_70_ and IC_80_ values for parent and ECF-R cancer cells. (D–F) 6 overlapping molecule protein levels in normal human tissue were acquired from a proteome database (“ProteomicsDB”). (D) Heatmap of the 6 overlapping molecules in various organs. Red arrow indicates protein levels of the 6 overlapping molecules in the stomach. (E and F) Median protein expression level for NINJ2 isoform-1 (Q9NZG7) and NINJ2 isoform-3 (F5H3L1).

**Figure S2**


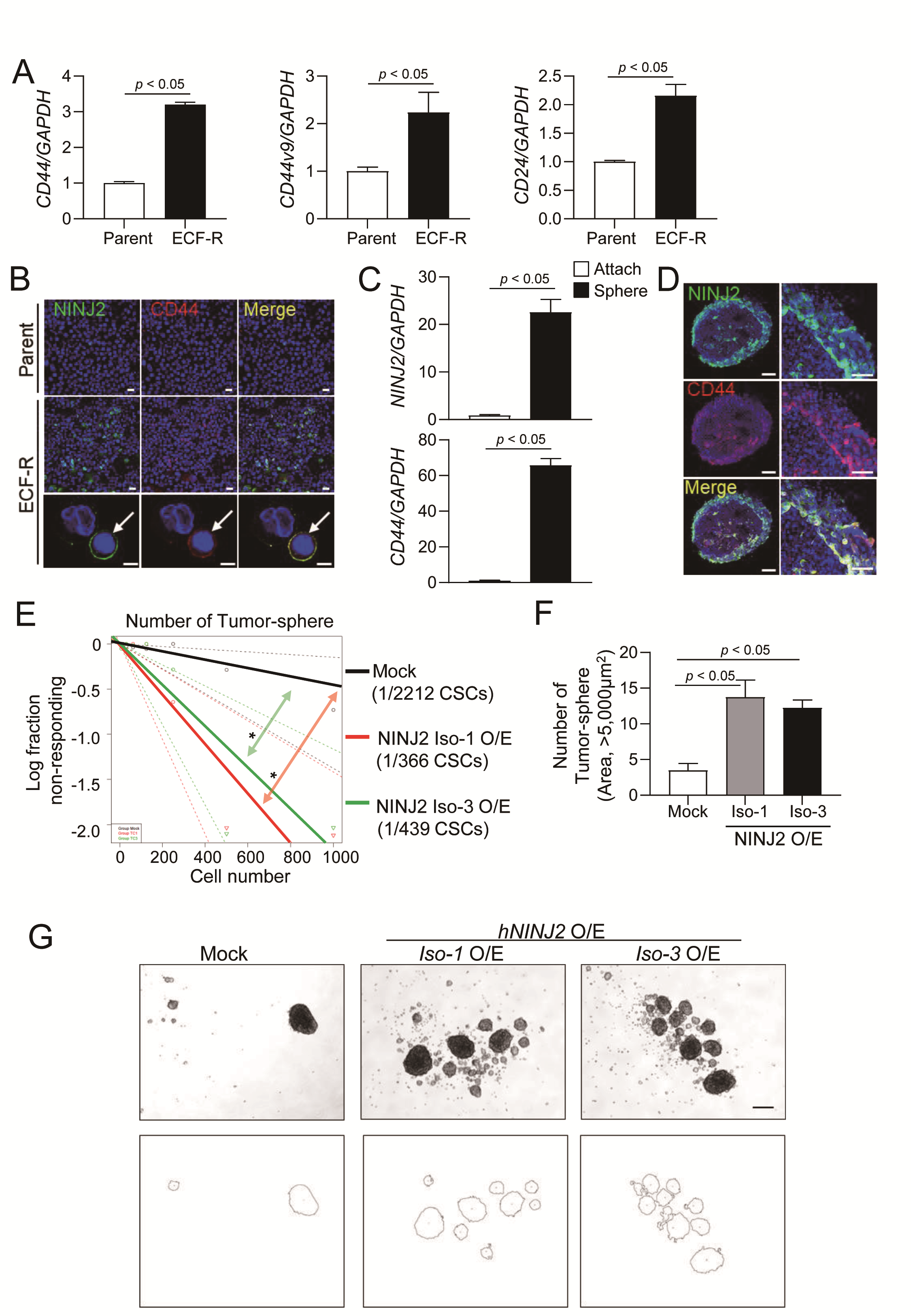


Figure S2. (A) mRNA level of CD44, CD44v9, and CD24 in parental and ECF-R MKN-74 cells. (B) Iummofluorescence staning images showing NINJ2 (green) and CD44 (red) expression in parent and ECF-R MKN-74 cancer cells. Scale bar, 20 µm. Arrow indicates NINJ2^+^CD44^+^ cells in ECF-R. Scale bar, 5 µm. (C) *NINJ2* or *CD44* mRNA level in MKN-74-derived tumorspheres. (D) NINJ2- and CD44-expressing cells in MKN-74-derived tumorsphere. Scale bar, 100 µm. High-magnification images of the boxed area in the figure. Scale bar, 50 µm. (E) Tumorsphere forming assay in MKN-74 cells stably overexpressing NINJ2. (F) Quantification of tumorspheres >5,000 µm^2^ per sphere was conducted using ImageJ. (G) (Top) Representative figures for tumorsphere-forming assay in stable NINJ2-overexpressing MKN-74 cells. (Bottom) Representative figures showing the outlines of spheres >5,000 µm^2^ for quantification.O/E, overexpression; Iso, isoform. Data are presented as mean ± SD.

**Figure S3**


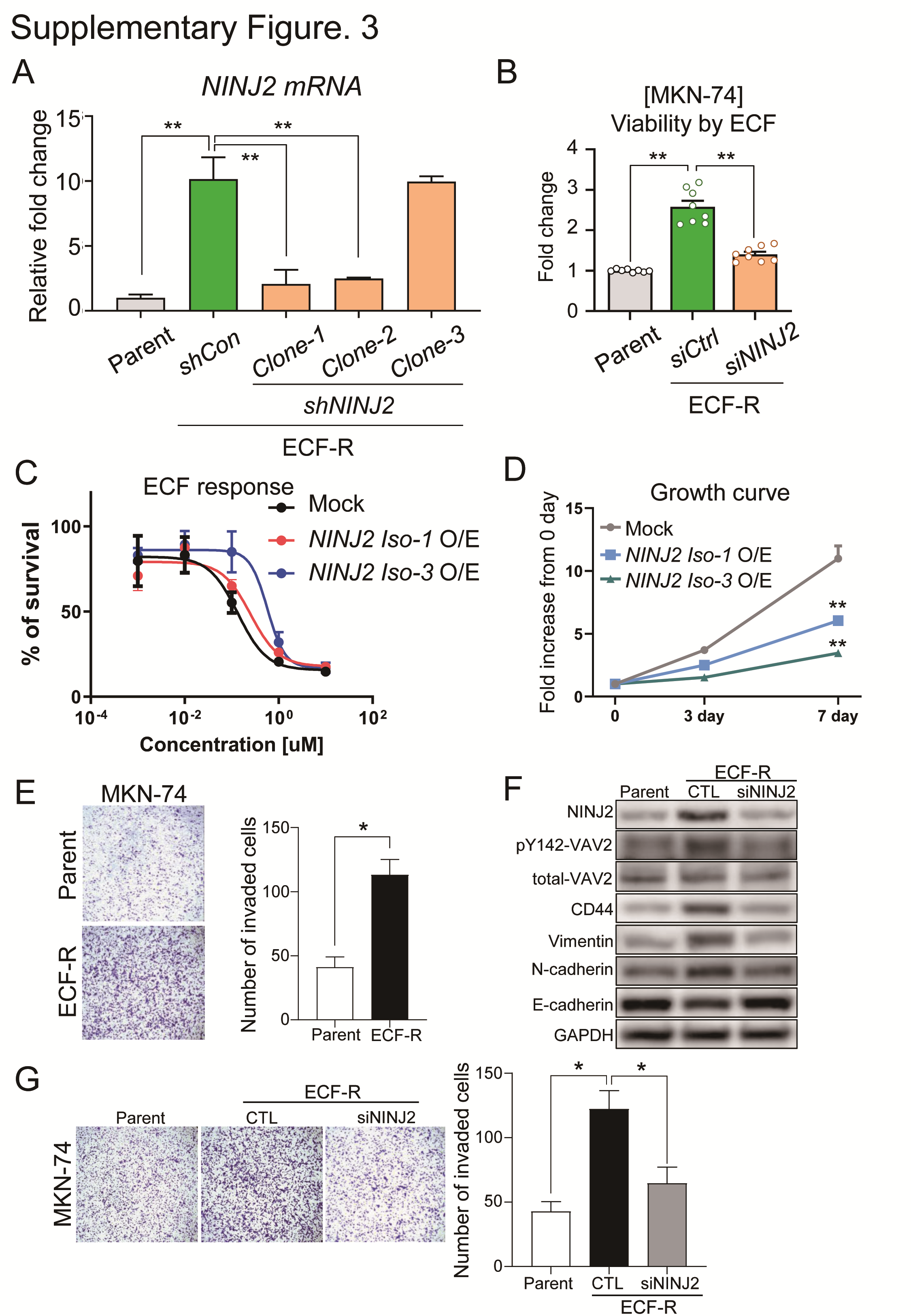


Figure S3. (A) ECF-R MKN-74 cells were transduced by lentiviral particles targeting different regions of the NINJ2 gene (isoform-1, isoform-2, and isoform-3) using TRCN0000063773 (clone 1), TRCN0000063775 (clone 2), and TRCN0000063776 (clone 3). Negative control was transduced by lentiviral particle with a non-targeting pLKO.1-puro shRNA control (SHC002). Lentiviral-transduced MKN-74 cells were analyzed for *NINJ2* knock-down using qRT-PCR. Among the three shRNA clones, clone 1 and clone 2 were highly effective and thus used for subsequent analyses. (B) Viability of 40–50 nM ECF in parent, ECF-R, and NINJ2 knock-down ECF-R (siRNA) MKN-74 cancer cells (N=3 biological replicates with at least 3 technical replicates per experiment, **P<0.05). (C) Representative image of four-parameter logistic curve for ECF in parental and ECF-R MKN-74 cells. (D) Cell proliferation analysis of mock and NINJ2 O/E MKN-74 cells using WST-8 on days 0, 3, and 7. (E) Invasion potential of cells in parent and ECF-R MKN-74 cancer cells (N=3 biological replicates with at least 3 technical replicates per experiment, *P<0.05). (F) Western blot analysis of NINJ2, pVAV2, tVAV2, CD44, vimentin, N-cadherin, E-cadherin, and GAPDH in parent, ECF-R, and NINJ2 knock-down ECF-R (siRNA) MKN-74 cancer cells (N=3 biological replicates with at least 3 technical replicates per experiment, **P<0.05). (G) Invasion potential of cells in parent, ECF-R, and NINJ2 knock-down ECF-R (siRNA) MKN-74 cancer cells (N=3 biological replicates with at least 3 technical replicates per experiment, *P<0.05). Data are presented as mean ± SD.

**Figure S4**


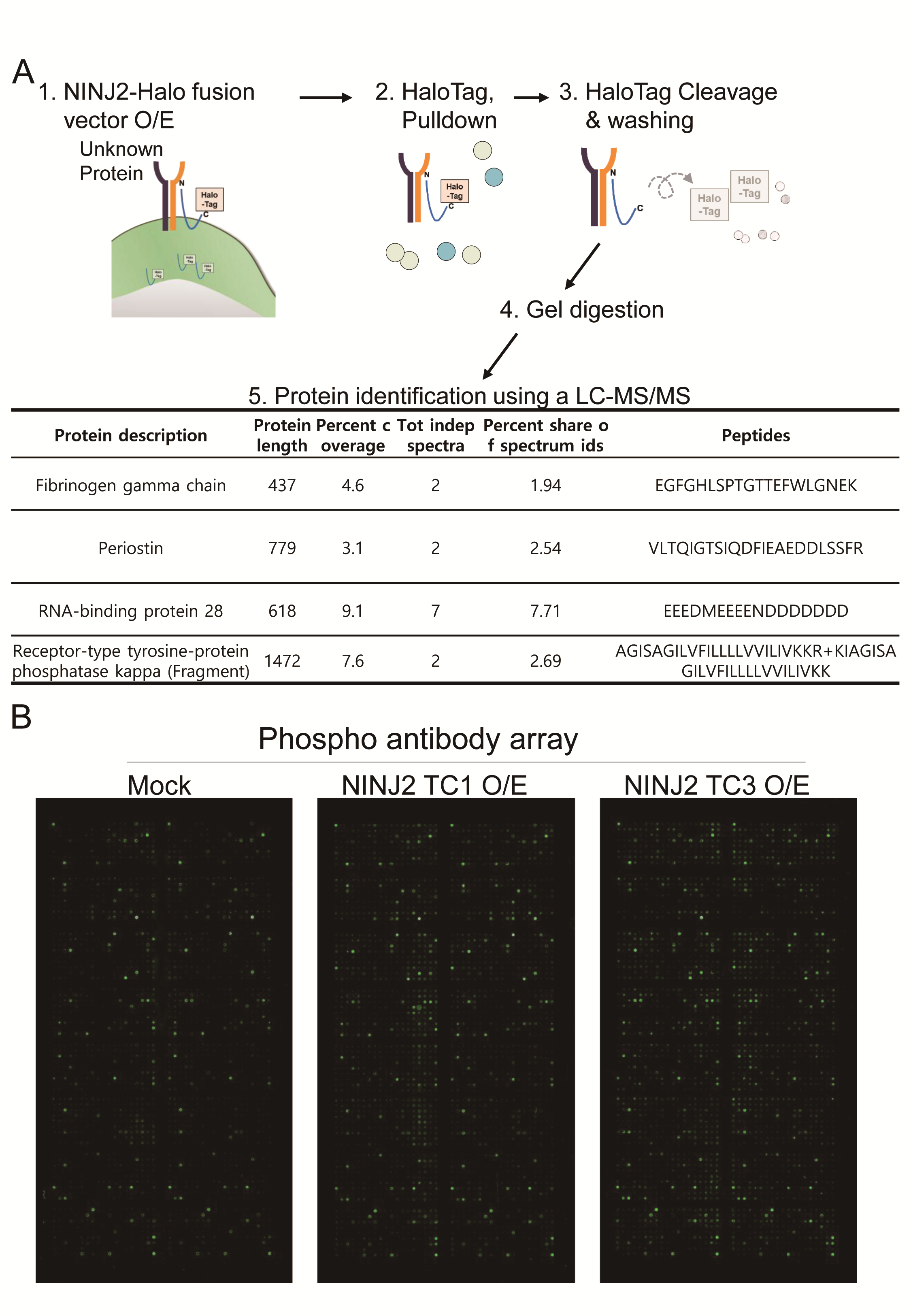


Figure S4. (A) Schematic representation of the experiment. NINJ2 binding molecules were identified by LC-MS/MS after pull-down of the NINJ2 complex. (B) Scanned image of phospho-protein antibody array containing 1,318 site-specific and phospho-specific antibodies (N=3 replicates per antibody).

**Figure S5**


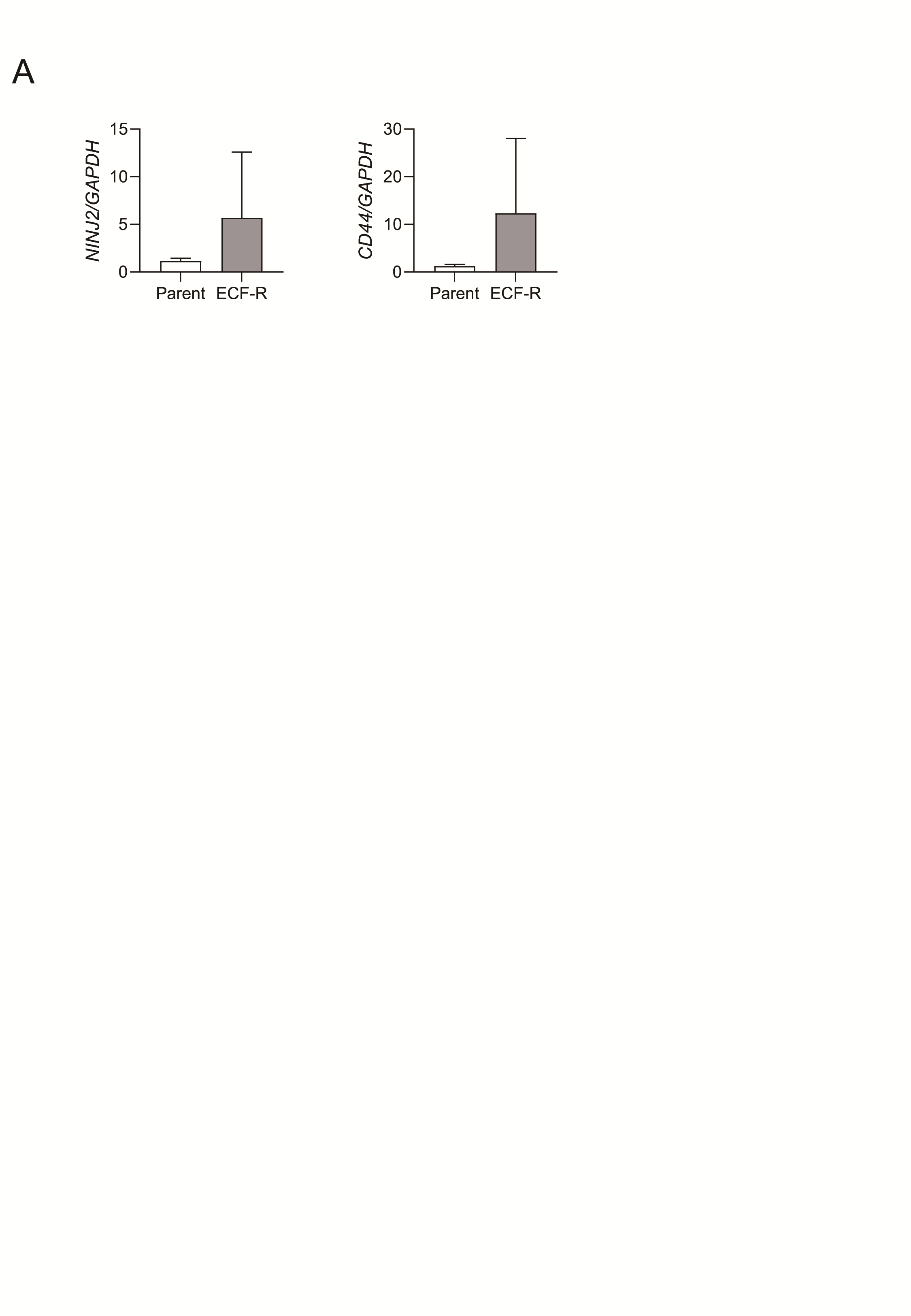


Figure S5. (A) mRNA level of *NINJ2* and *CD44* in parental and ECF-R MKN-74 tumors. Data are presented as mean ± SD.

**Figure S6**


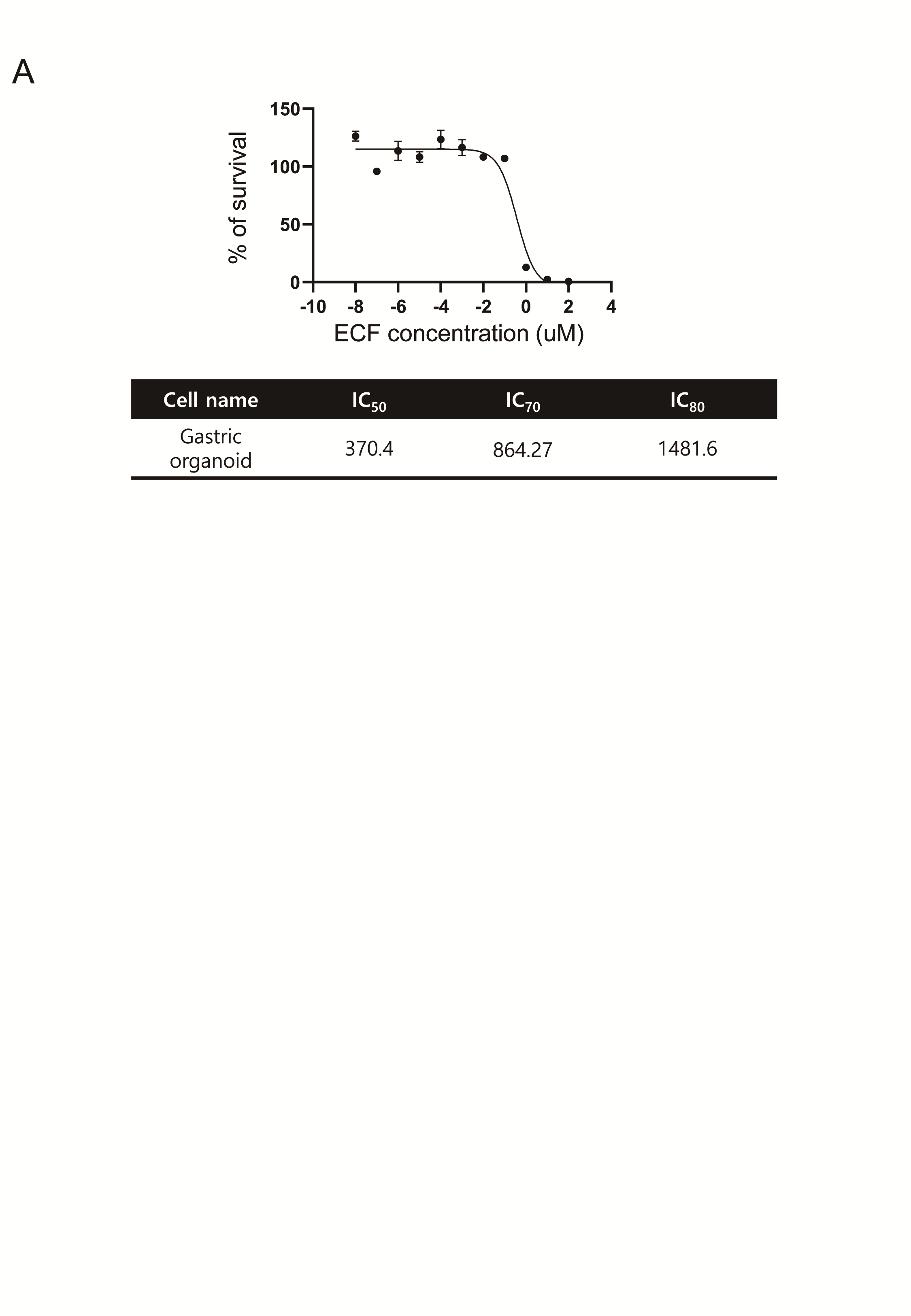


Figure S6. (A) (Top) ECF IC_50_ values for patient-derived gastric tumor organoids. (Bottom) Table of IC_50_, IC_70_, and IC_80_ values for gastric tumor organoids. Data are presented as mean ± SD.
